# A colorimetric approach for monitoring the reduction of platinum(iv) complexes in aqueous solution†

**DOI:** 10.1039/d4nj00859f

**Published:** 2024-04-10

**Authors:** Shitong Huang, Jevon W. Marsh, Jhanelle R. G. White, Tracy Q. Ha, Sophie A. Twigger, Ismael Diez-Perez, Adam C. Sedgwick

**Affiliations:** a Chemistry Research Laboratory, University of Oxford Mansfield Road OX1 3TA UK adam.sedgwick@kcl.ac.uk; b Department of Oncology, University of Oxford Old Road Campus Research Building Oxford OX3 7DQ UK; c Department of Chemistry, King's College London 7 Trinity Street London SE1 1DB UK

## Abstract

We report the synthesis of 4-nitrophenyl (4-NP) functionalised Pt(iv) complexes as a colorimetric strategy for monitoring Pt(iv) reduction in aqueous solution. Treatment of each 4-NP functionalised Pt(iv) complex with the biological reductant sodium ascorbate led to a colour change from clear to yellow, which was attributed to the reduction of Pt(iv) to Pt(ii) and simultaneous release of 4-nitroaniline. Trends in reduction profiles and a photocatalysed reduction for each Pt(iv) complex were observed.

## Abstract 2

We report the synthesis of 4-nitrophenyl (4-NP) functionalised Pt(iv) complexes as a colorimetric strategy for monitoring Pt(iv) reduction in aqueous solution.

## Graphical abstract

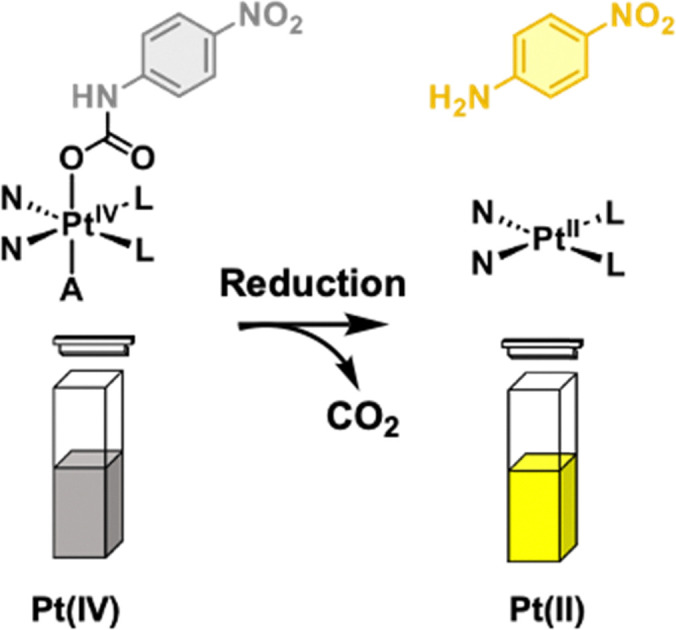

Cisplatin, oxaliplatin, and carboplatin are amongst the most widely used chemotherapeutics in oncology.^1^ Unfortunately, platinum(ii) anticancer agents are plagued by severe toxicity issues, which ultimately impact treatment success rates.^2^ In an attempt to address these limitations, efforts have been devoted to studying the Pt(iv) analogues of these FDA-approved chemotherapeutics.^3^ The kinetic inertness of these low-spin, octahedral Pt(iv) complexes has the potential to reduce off-target toxicity and enhance drug bioavailability.^4,5^ Our current understanding is that Pt(iv) complexes undergo cancer selective reduction and form a toxic Pt(ii) product with the loss of two axial ligands.^1^ Gibson and co-workers highlighted that we cannot eliminate the possibility of forming more than one Pt(ii) species.^6,7^ Although promise has been shown in preclinical studies, no Pt(iv) anticancer agent has been approved.^8,9^ One of the biggest obstacles to Pt(iv) therapy is the need to improve our understanding of Pt(iv) reduction and identify how it correlates within a biological context.^10,11^ Several research groups have developed diagnostic strategies to assist in these efforts.^12–14^ Here, we present a simple colorimetric strategy that allows monitoring of the reduction of Pt(iv) to Pt(ii) in aqueous solution to help identify factors that lead to reduction, Scheme 1.

**Scheme 1:**
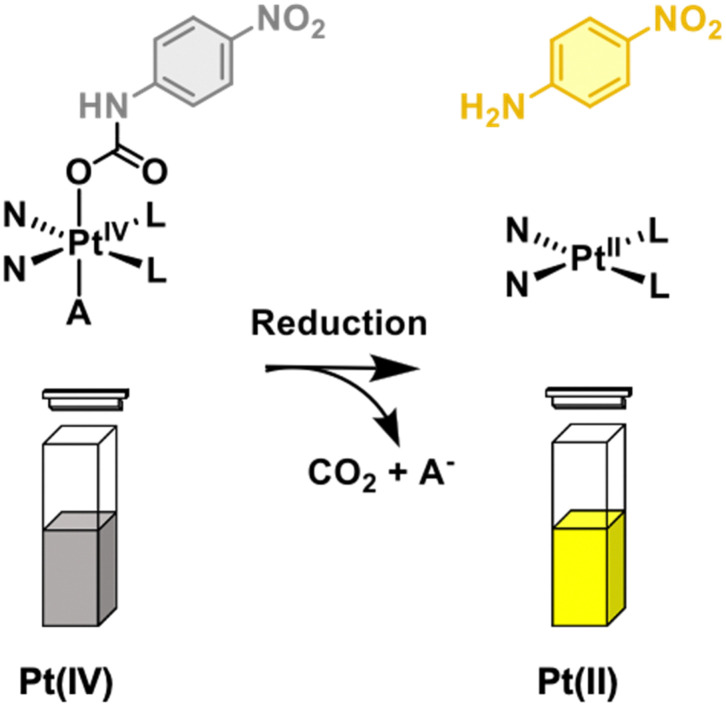
The general design of the colorimetric strategy developed in this study for monitoring platinum(iv) reduction.

The most routine method for studying both the stability and reduction of Pt(iv) complexes is through high-performance liquid chromatography (HPLC).^15^ Although effective, most Pt-complexes are weakly absorbing, resulting in the need of millimolar concentrations for each complex to be seen on the HPLC chromatogram. In addition, the long wait times between HPLC experiments prevents the rapid screening of potential reductants. Optical techniques such as UV-Vis spectroscopy have found widespread use in assay development including high-throughput screening.^11–13^ We rationalised by installing 4-nitroaniline onto the axial position of a Pt(iv) complex, we could access a general approach to screen reductants and study the reduction of Pt(iv) to Pt(ii) by UV-Vis spectroscopy. With this design, Pt(iv) reduction results in the release of free strongly absorbing 4-nitroaniline (yellow colour, *λ*_max_ = 385 nm, see ESI,† Fig. S1) *via* rapid decarboxylation of an unstable carbamic acid intermediate (Scheme 1).^15–17^ We believe this strategy would help improve our efforts in identifying factors that lead to the reduction of Pt(iv) to Pt(ii).

As a proof of concept, 4-nitrophenyl (4-NP) functionalised Pt(iv) complexes (Cis-4NP, Oxali-4NP and Carbo-4NP) were synthesised, derived from the classical platinum anticancer agents, cisplatin(ii), oxaliplatin(ii) and carboplatin(ii) (Fig. 1). Each Pt(ii) complex was first oxidised to the corresponding Pt(iv) hydroxo-aceto complex, CisPt(OH)(OAc) (1), OxPt(OH)(OAc) (2), and CbPt(OH)(OAc) (3) using hydrogen peroxide and acetic acid as a solvent (see ESI,† Scheme S1). Subsequent treatment of each Pt(iv) reagent (1–3) with 4-nitrophenylisocyanate in DMF afforded the desired 4-NP functionalised Pt(iv) complexes, Cis-4NP, Oxali-4NP and Carbo-4NP in yields of 30%, 44% and 46%, respectively (see ESI,† Scheme S2 and for the full experimental methods).^14^

**Fig. 1:**
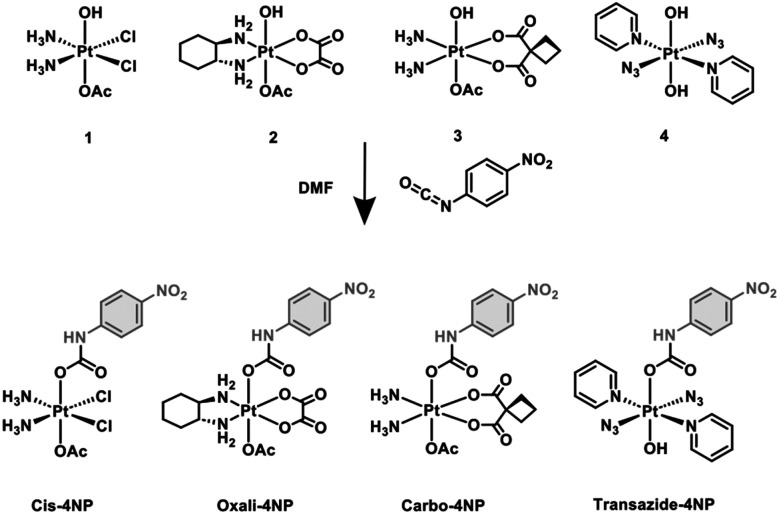
Reaction scheme for the synthesis of 4-nitrophenyl functionalised Pt(iv) complexes used in this study (Cis-4NP, Oxali-4NP, Carbo-4NP and Transazide-4NP).

With the 4-NP functionalised Pt(iv) complexes in hand, we turned our attention to evaluating the photophysical and stability properties of each complex in solution (PBS, pH = 7.40). All three Pt(iv) complexes displayed strong absorption bands centred at ∼340 nm and were found stable over the course of 24 hours (see ESI,† Fig. S2–S4). HPLC analysis was used as complementary method to confirm the stability of each complex over a 24-hour period (see ESI,† Fig. S5–S7). The concentrations needed for UV-Vis spectroscopy was also shown suitable for HPLC analysis; no HPLC trace of 2 was observed at the same concentrations, highlighting our inability to often study Pt(iv) complexes at biologically relevant concentrations (see ESI,† Fig. S8). Sodium ascorbate (NaAsc) is a routinely used biological reductant for Pt(iv) chemistry.^1,15^ Excitingly, the addition of NaAsc (100 equiv., 5 mM) to a solution of Cis-4NP (50 μM), led to a gradual change in UV-Vis absorption over the course of 7 hours with a decrease at 338 nm and increase at 404 nm (Fig. 2). Observable to the naked eye was a colour change from clear to a bright yellow solution indicative of the release of 4-nitroaniline (see ESI,† Fig. S9). The release of 4-nitroaniline was confirmed by HPLC (see ESI,† Fig. S10). Interestingly, when compared to Oxali-4NP and Carbo-4NP, and in line with previous reports,^15^ a trend in NaAsc sensitivity was observed (Cis-4NP > Oxali-4NP > Carbo-4NP). As seen in Fig. 2c and d, Oxali-4NP and Carbo-4NP treated with NaAsc (100 equiv. 5 mM) displayed smaller changes to the corresponding absorption spectra and HPLC traces when compared to the untreated controls (see ESI,† Fig. S11 and S12). Mass spectrometry confirmed the reduction of Oxali-4NP and Carbo-4NP to oxaliplatin and carboplatin, respectively. Whereas, Cis-4NP afforded a mixture of Pt(ii) species (see ESI,† Fig. S13–S15). The response of each 4-NP complex was shown selective for NaAsc when compared to other biological reductants such as glutathione (GSH) and l-cysteine (l-Cys) (Fig. 2; see ESI† for spectra, Fig. S16–S18). Overall, the obtained data confirms the success of this colorimetric strategy.

**Fig. 2:**
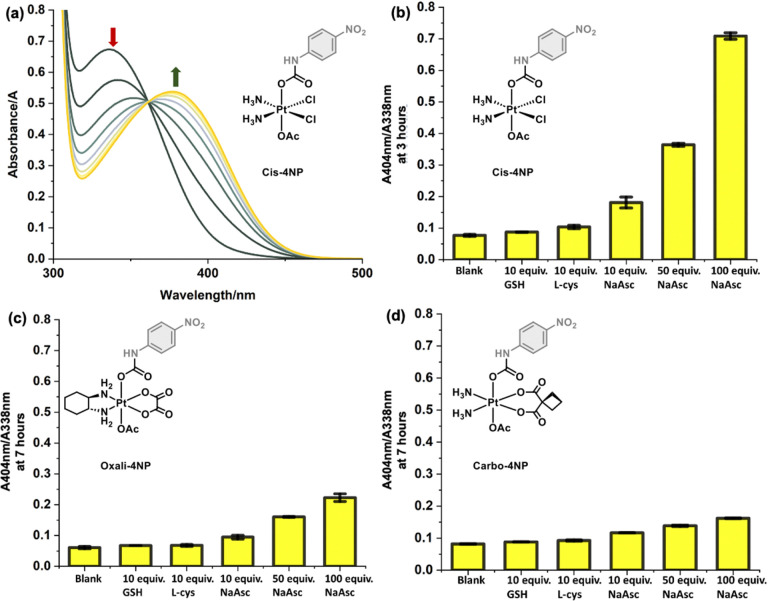
(a) Absorption spectra of Cis-4NP (50 μM) in a PBS buffer solution (pH = 7.40, with 1% DMF) containing NaAsc (100 equiv., 5 mM). Measurements were taken over the course of 7 hours. (b) Bar chart of the change in absorption (404 nm/338 nm) of Cis-4NP (50 μM) treated with different reductants NaAsc (100/50/10 equiv., 5/2.5/0.5 mM), l-cys (10 equiv., 0.5 mM) and GSH (10 equiv., 0.5 mM) and measured after 3 hours incubation. (c) and (d) Bar charts of the changes in absorption (404 nm/338 nm) of Oxali-4NP and Carbo-4NP (50 μM) treated with different reductants; NaAsc (100/50/10 equiv., 5/2.5/0.5 mM); l-cys (10 equiv., 0.5 mM) and GSH (10 equiv. 0.5 mM) and measured after 7 hours incubation. Error bars represent SD.

To demonstrate the general utility of this approach, we wanted to apply this strategy to a non-classical Pt(iv) complex. *t*,*t*,*t*-[Pt(N_3_)_2_(OH)_2_(py)_2_] (transazide) was reported in 2010 as a photoactivatable chemotherapeutic (PACT), only displaying cytotoxicity upon light irradiation.^18,19^ It is believed that Transazide forms reactive azide-based radicals under light irradiation, which leads to the formation of a mixture of cytotoxic Pt(iv) and Pt(ii) species.^18,20^ Using the same synthetic protocols, we isolated Transazide-4NP in 57% yield (see ESI,† Scheme S2). Determined by UV-Vis and HPLC, Transazide-4NP displayed excellent aqueous stability over the course of 24 hours (see ESI,† Fig. S19 and S20). Confirming the ability to use our colorimetric strategy for this light activatable Pt(iv) complex, Transazide-4NP was irradiated for 5 hours using a desktop lamp fitted with a Deltech LED bulb (wavelength: 350-400 nm, 80 Lumen, 1.2 W) (see ESI,† General information and methods section). As seen in Fig. 3a, under light irradiation, a gradual change in absorption spectra was observed with a decrease in 338 nm and increase in absorption at 404 nm. The release of 4-nitroaniline from Transazide-4NP was confirmed by HPLC and NMR analysis (see ESI,† Fig. S21 and S22). More than one peak was observed in the HPLC chromatogram post light irradiation, suggesting the formation of several Pt-based species as described in previous reports.^18^ As a comparison, we wanted to test how each of the classical Pt(iv) complexes Cis-4NP, Oxali-4NP and Carbo-4NP behaved under the same light irradiation conditions to Transazide-4NP. Previous efforts by the groups of Ang and Zhu have reported the design of light responsive Pt(iv) complexes that are based on the FDA-approved scaffolds, thus warranting the exploration of our colorimetric systems.^21,22^ To our surprise, the light irradiation of Cis-4NP resulted in a rapid change to its absorption spectra, which was at a similar rate to the light activation of Transazide-4NP (see ESI,† Fig. S23 and S24). In stark contrast, minimal changes to the absorption spectra of Oxali-4NP and Carbo-4NP was observed after 3 hours of light irradiation (see ESI,† Fig. S23 and S24). Previous reports have shown NaAsc as a potential catalyst for the photoreduction of Pt(iv) complexes,^18,19^ we were therefore motivated to test the influence of NaAsc on the rate of the photo-mediated reduction of Cis-4NP, Oxali-4NP and Carbo-4NP. Remarkably, the presence of NaAsc during the light irradiation experiments resulted in significant increases in the rate of change of the absorption spectra of Cis-4NP, Oxali-4NP and Carbo-4NP, Fig. 3b–d. The influence of NaAsc and light proved greater than either NaAsc or light alone for each complex (see ESI,† Fig. S25–S230), which was also confirmed by HPLC analysis (see ESI,† Fig. S31–S33). Unexpectedly, the addition of NaAsc (100 equiv., 2.5 mM) to a solution of Transazide-4NP (25 μM) resulted in a change to the absorption spectra along with the observed release of 4-nitroaniline, which suggests the reduction of Pt(iv) to Pt(ii) (see ESI,† Fig. S34–S36). To the best of our knowledge this is an observation not previously reported.

**Fig. 3:**
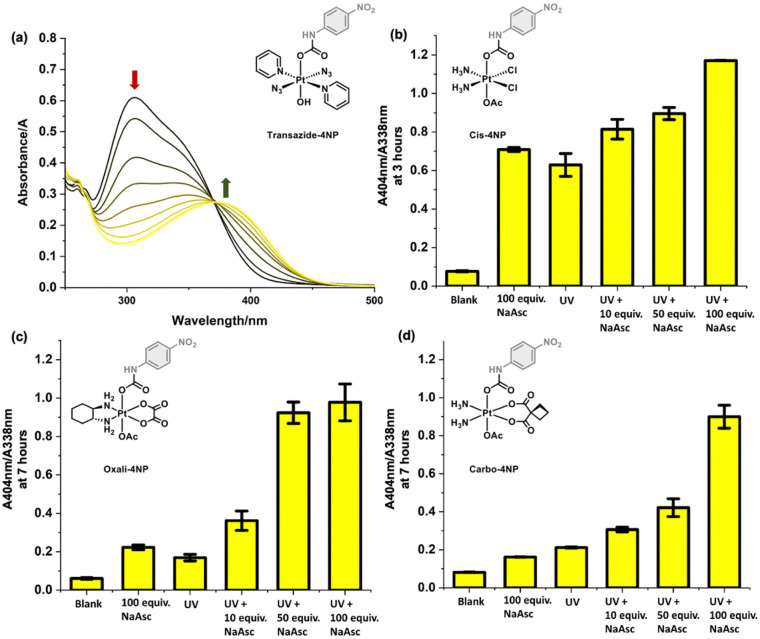
(a) Absorption spectra of Transazide-4NP (25 μM) in PBS buffer solution (pH = 7.40, with 1% DMF) under light irradiation from 0–5 hours. (b) Bar chart of the change in absorption (A404 nm/A338 nm) of Cis-4NP with difference concentrations of NaAsc (100/50/10 equiv., 5/2.5/0.5 mM) under light irradiation (3 hours). (c) and (d) Bar chart of the change in absorption (*A*_404nm_/*A*_338nm_) of Oxali-4NP/Carbo-4NP (50 μM) with difference concentrations of NaAsc (NaAsc (100/50/10 equiv., 5/2.5/0.5 mM) under light irradiation (7 hours)). Error bars represent SD.

Throughout this study, we have observed a trend in the sensitivity of NaAsc (and photo-)mediated reduction for the classical platinum agents Cis-4NP > Oxali-4NP > Carbo-4NP. To rationalise this trend, we wanted to interpret the chemical environment of the Pt centre by ^195^Pt NMR and cyclic voltammetry. Use of ^195^Pt NMR revealed distinctly different chemical shifts between Cis-4NP, Oxali-4NP and Carbo-4NP, with values of 1253 ppm, 1632 ppm and 1971 ppm, respectively. The NMR information obtained suggests the strong influence of the equatorial ligands towards Pt(iv) reduction. Further supporting this observation, cyclic voltammetry shows increase (becoming more negative) in reduction potential from Cis-4NP to Carbo-4NP (−0.25/−0.30/−0.38 V *vs*. Ag/AgCl see ESI,† Fig. S37 and S38) indicating that carboplatin-based Carbo-4NP is least sensitive to reduction as also shown *via* our colorimetric strategy.

In summary, we report 4-nitrophenyl functionalised Pt(iv) complexes as a colorimetric strategy to provide an optical method for monitoring Pt(iv) reduction. 4-NP functionalised Pt(iv) complexes of the classical platinum anticancer agents, cisplatin(ii), oxaliplatin(ii) and carboplatin(ii) were synthesised. NaAsc-mediated reduction was monitored *via* UV-Vis spectroscopy, which identified trends in NaAsc sensitivity between complexes. An unexpected photocatalysed reduction of Pt(iv) complexes was observed. We believe this simple colorimetric strategy has the potential to find future utility in other research labs for studying the reduction of Pt(iv) complexes without solely relying on HPLC instrumentation.

## Conflicts of interest

There are no conflicts to declare.

## Supplementary Material

NJ-048-D4NJ00859F-s001

## References

[cit1] Johnstone T. C., Suntharalingam K., Lippard S. J. (2016). Chem. Rev..

[cit2] Stojanovska V., Sakkal S., Nurgali K. (2015). Am. J. Physiol.: Gastrointest. Liver Physiol..

[cit3] Xu Z., Wang Z., Deng Z., Zhu G. (2021). Coord. Chem. Rev..

[cit4] Gibson D. (2019). J. Inorg. Biochem..

[cit5] Najjar A., Rajabi N., Karaman R. (2017). Curr. Pharm. Des..

[cit6] Wexselblatt E., Gibson D. (2012). J. Inorg. Biochem..

[cit7] Nemirovski A., Vinograd I., Takrouri K., Mijovilovich A., Rompel A., Gibson D. (2010). Chem. Commun..

[cit8] Thiabaud G., He G., Sen S., Shelton K. A., Baze W. B., Segura L., Alaniz J., Munoz Macias R., Lyness G., Watts A. B., Kim H. M., Lee H., Cho M. Y., Hong K. S., Finch R., Siddik Z. H., Arambula J. F., Sessler J. L. (2020). Proc. Natl. Acad. Sci. U. S. A..

[cit9] Wheate N. J., Walker S., Craig G. E., Oun R. (2010). Dalton Trans..

[cit10] Boulet M. H. C., Bolland H. R., Hammond E. M., Sedgwick A. C. (2023). J. Am. Chem. Soc..

[cit11] Yao H., Zhu G. (2022). Dalton Trans..

[cit12] Ong J. X., Lim C. S. Q., Le H. V., Ang W. H. (2019). Angew. Chem., Int. Ed..

[cit13] New E. J., Duan R., Zhang J. Z., Hambley T. W. (2009). Dalton Trans..

[cit14] Liu G., Zhang Y., Yao H., Deng Z., Chen S., Wang Y., Peng W., Sun G., Tse M.-K., Chen X., Yue J., Peng Y.-K., Wang L., Zhu G. (2023). Sci. Adv..

[cit15] Chen S., Yao H., Zhou Q., Tse M. K., Gunawan Y. F., Zhu G. (2020). Inorg. Chem..

[cit16] Babu T., Sarkar A., Karmakar S., Schmidt C., Gibson D. (2020). Inorg. Chem..

[cit17] Carl P. L., Chakravarty P. K., Katzenellenbogen J. A. (1981). J. Med. Chem..

[cit18] Farrer N. J., Woods J. A., Salassa L., Zhao Y., Robinson K. S., Clarkson G., Mackay F. S., Sadler P. J. (2010). Angew. Chem., Int. Ed..

[cit19] Farrer N. J., Salassa L., Sadler P. J. (2009). Dalton Trans..

[cit20] Bolitho E. M., Sanchez-Cano C., Shi H., Quinn P. D., Harkiolaki M., Imberti C., Sadler P. J. (2021). J. Am. Chem. Soc..

[cit21] Lee V. E. Y., Chin C. F., Ang W. H. (2019). Dalton Trans..

[cit22] Yao H., Chen S., Deng Z., Tse M.-K., Matsuda Y., Zhu G. (2020). Inorg. Chem..

