# Supplementary material for: A colorimetric approach for monitoring the reduction of platinum(iv) complexes in aqueous solution
Source: New J Chem. 2024 Apr 10;48(17):7548–51. doi: 10.1039/d4nj00859f (PMC11057408; doi:10.1039/d4nj00859f)
Supplement: NJ-048-D4NJ00859F-s001 [file NJ-048-D4NJ00859F-s001.pdf]

Electronic Supplementary Material (ESI) for New Journal of Chemistry.
This journal is © The Royal Society of Chemistry and the Centre National de la Recherche Scientifique 2024

Supporting Information

A colorimetric approach for monitoring platinum(IV) reduction in aqueous solution

Shitong Huang,[a] Jevon W. Marsh,[a] Jhanelle R. G. White,[c] Tracy Q. Ha,[c] Sophie A. Twigger,[b] Ismael Diez-Perez,[c] and Adam C. Sedgwick[a,c*]

[a]Chemistry Research Laboratory, University of Oxford, Mansfield Road, OX1 3TA, United Kingdom

[b]Department of Oncology, University of Oxford, Old Road Campus Research Building, Oxford, OX3 7DQ, United Kingdom

[c]Department of Chemistry, King's College London, 7 Trinity Street, London, SE1 1DB, United Kingdom

Email: adam.sedgwick@kcl.ac.uk

Table of Contents

1. General Information and Methods……………………………..S3

2. Synthetic Schemes……………………..……………………………….S5

3. Analytical Experiments………………..…………………………….S6

4. Synthetic procedures...S37

5. NMR Spectra S42

6. References S62

1. General Information and Methods

Materials and Chemicals

All chemicals and reagents were purchased commercially and were of analytical grade. UV absorption spectra were obtained on a Jasco V-770 UV-Visible/NIR Spectrophotometer using quartz cuvettes of 1 cm path length. Light irradiation experiments were performed using a Deltech 1.2W LED GU10 PAR16 UV-DL-9021 UV LED bulbs (80 Lumen Wavelength: 350-400nm). Column chromatography was carried out using Merck® silica gel 60 under a positive pressure of nitrogen. Eluent ratios are reported by volume percentages. NMR spectra were recorded on a Bruker AVIII 400, Bruker AVII 500 (with cryoprobe) and Bruker AVIII 500 spectrometers. Chemical shifts are reported as δ values in ppm. Mass spectra were performed using Thermo Exactive High-Resolution Orbitrap FTMS. All water used in analytical experiments was Milli-Q grade (18.2 MΩ, Millipore). HPLC analysis was carried out using Thermo scientific Vanquish HPLC system with Discovery Cyano column [5 µm, 4.6mm x 25cm]; HPLC conditions: 1µL/min, 0-35 min, MeCN:H_2O. For UV-Vis experiments, **Cis-4NP, Oxali-4NP, Carbo-4NP** and **Transazide-4NP** were first each dissolved in DMF to prepare a series of stock solutions (5mM). The stock solution was subsequently diluted in PBS buffer (pH = 7.40) to afford a working solution of 50 µM (25 µM solution of **Transazide-4NP**). The solutions were stored and measured at room temperature.

Light Irradiation Experiments

Cis-4NP, Oxali-4NP, Carbo-4NP and **Transazide-4NP** were first each dissolved in DMF to prepare a series of stock solutions (5mM). The stock solution was subsequently in PBS buffer (pH = 7.40) to make 50 µM solution of each complex (25 µM solution of **Transazide-4NP**). The sample solution in a quartz cuvette was placed 10 cm away from the light source. The sample was then irradiated using a UV-LED light bulb (Deltech 1.2W LED GU10 PAR16 UV-DL-9021 UV LED bulbs 80 Lumen Wavelength: 350-400nm). The photoreduction was monitored by UV-Vis Spectroscopy at different timepoints. Note – the same solutions (prepared in Milli Q water instead of PBS buffer) were used for HPLC analysis, where 40 µL of the sample was injected into the HPLC at different timepoints.

Electrochemistry Experiments

Cyclic voltammograms of all complexes were obtained using an Autolab IMP potentiostat (Metrohm, Netherlands) and three-electrode configuration, with a 3 mm glassy carbon working electrode, platinum wire as counter electrode, and a Basi Ag/AgCl (3 M KCl) reference electrode. The glassy carbon electrode was polished using 1 µm alumina slurry and sonicated in Milli-Q water (18.2 MΩ, Millipore) before immediate transferal to the electrolyte solution of 1 mM of **Cis-4NP**, **Oxali-4NP**, or **Carbo-4NP** in PBS buffer (pH 7.40) solution containing DMF (10 %). All electrolyte solutions were deaerated with nitrogen prior to each measurement, with solutions maintained at room temperature and under a stream of nitrogen throughout each measurement.

2. Synthetic Schemes

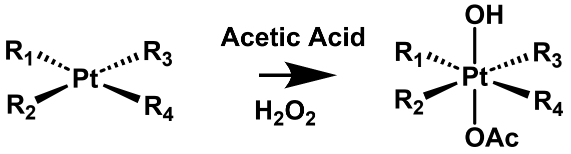

Complex 1: R_1=R_2= NH_3, R_3=R_4= Cl
Complex 2: R_1=R_2= (R,R)-DACH, R_3=R_4= oxalate
Complex 3: R_1=R_2=NH_3 , R_3=R_4= CBDCA

Scheme S1. General reaction scheme for the oxidation of each Pt(II) complex to the corresponding Pt(IV) reagent.

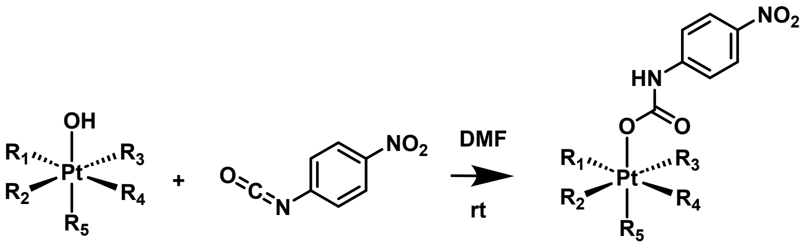

Complex 1: R_1=R_2= NH_3, R_3=R_4= Cl, R_5= OAc
Complex 2: R_1=R_2= (R,R)-DACH, R_3=R_4= oxalate, R_5= OAc
Complex 3: R_1=R_2=NH_3 , R_3=R_4= CBDCA, R_5= OAc
Complex 4: R_1=R_4=N_3 , R_2=R_3= pyridine, R_5= OH

Cis-4NP: R_1=R_2= NH_3, R_3=R_4= Cl, R_5= OAc
Oxali-4NP: R_1=R_2= (R,R)-DACH, R_3=R_4= oxalate, R_5= OAc
Carbo-4NP: R_1=R_2=NH_3 , R_3=R_4= CBDCA, R_5= OAc
Transazide-4NP:R_1=R_4=N_3 , R_2=R_3= pyridine, R_5= OH

Scheme S2. Synthetic scheme for the synthesis of **Cis-4NP**, **Oxali-4NP**, **Carbo-4NP** and **Transazide-4NP**

3. Additional Analyses

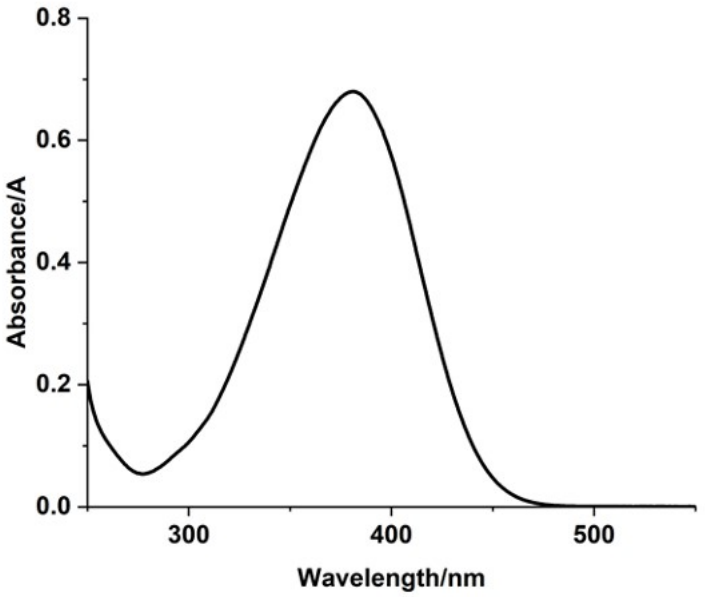

Figure S1. Absorption spectrum of **4-Nitroaniline** (50 µM) in PBS buffer (pH 7.40, with 1% DMF).

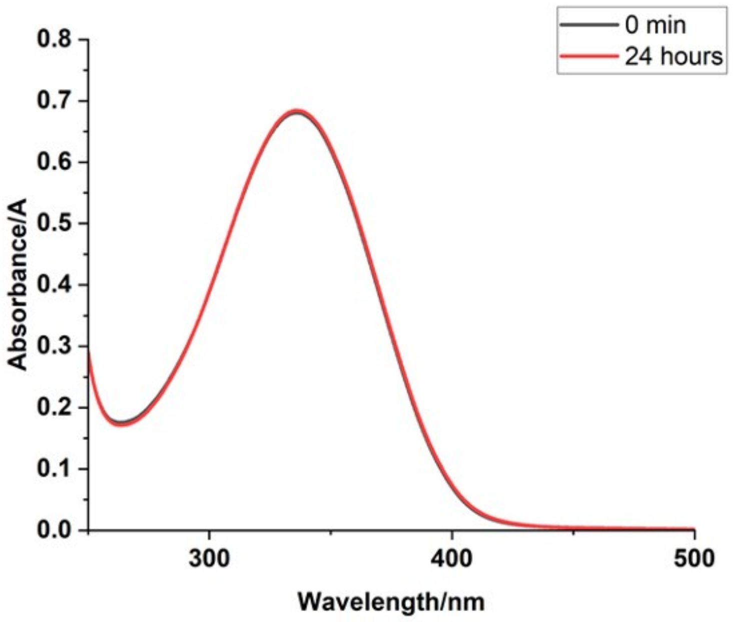

Figure S2. Absorption spectra of **Cis-4NP** (50 µM) in PBS buffer (pH 7.40, with 1 % DMF) at 0 and 24 hours.

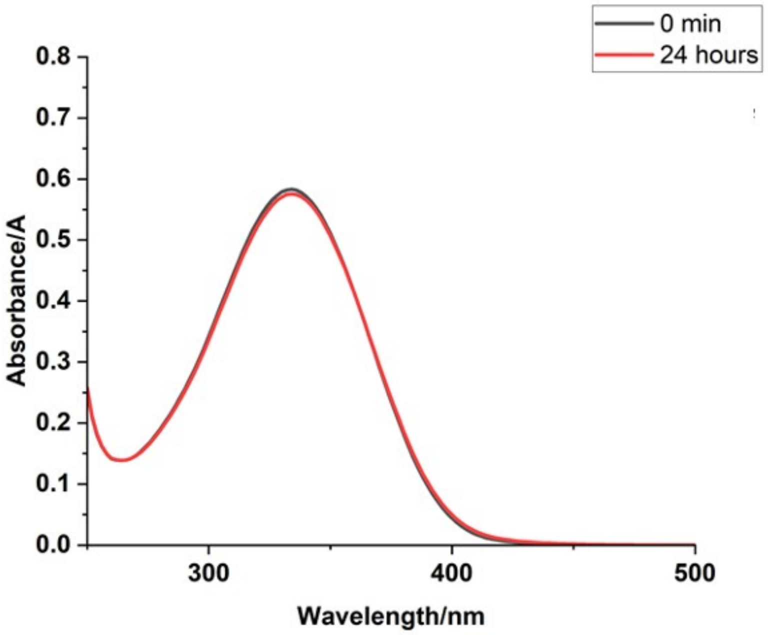

Figure S3. Absorption spectra of **Oxali-4NP** (50 µM) in PBS buffer (pH 7.40, with 1% DMF) at 0 and 24 hours.

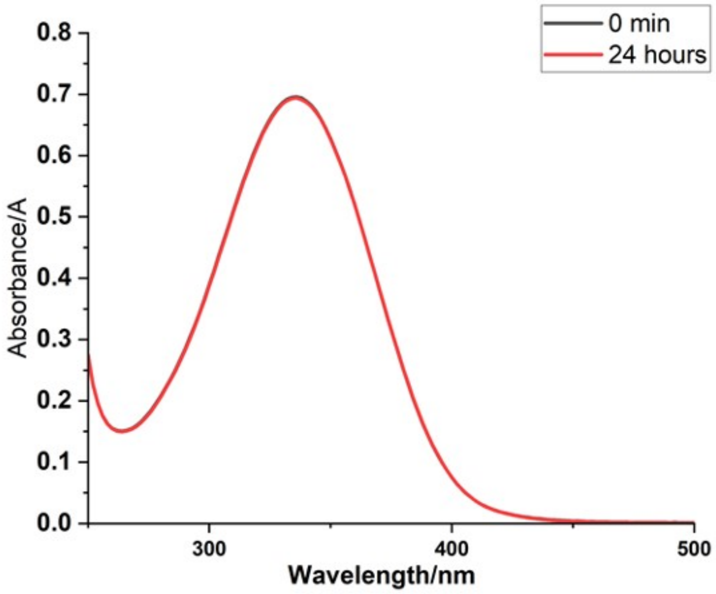

Figure S4. Absorption spectra of **Carbo-4NP** (50 µM) in PBS buffer (pH 7.40, with 1% DMF) at 0 and 24 hours.

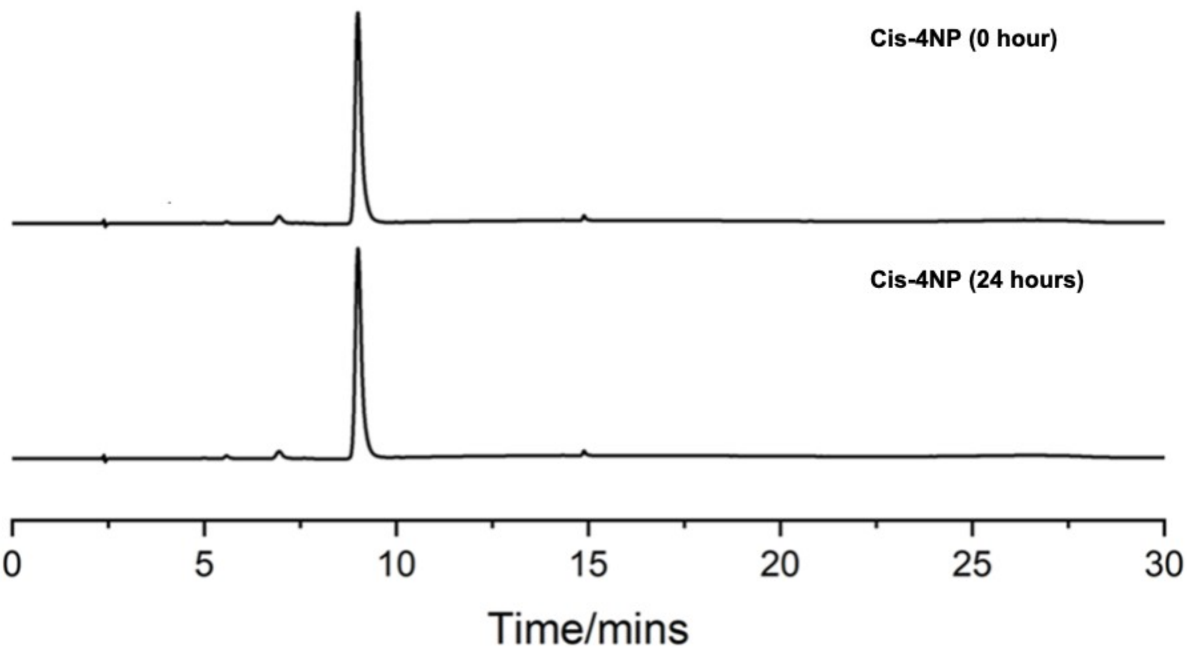

Figure S5. HPLC traces of **Cis-4NP** (50 µM) in water (with 1% DMF) at 0 and 24 hours (absorption at 340 nm).

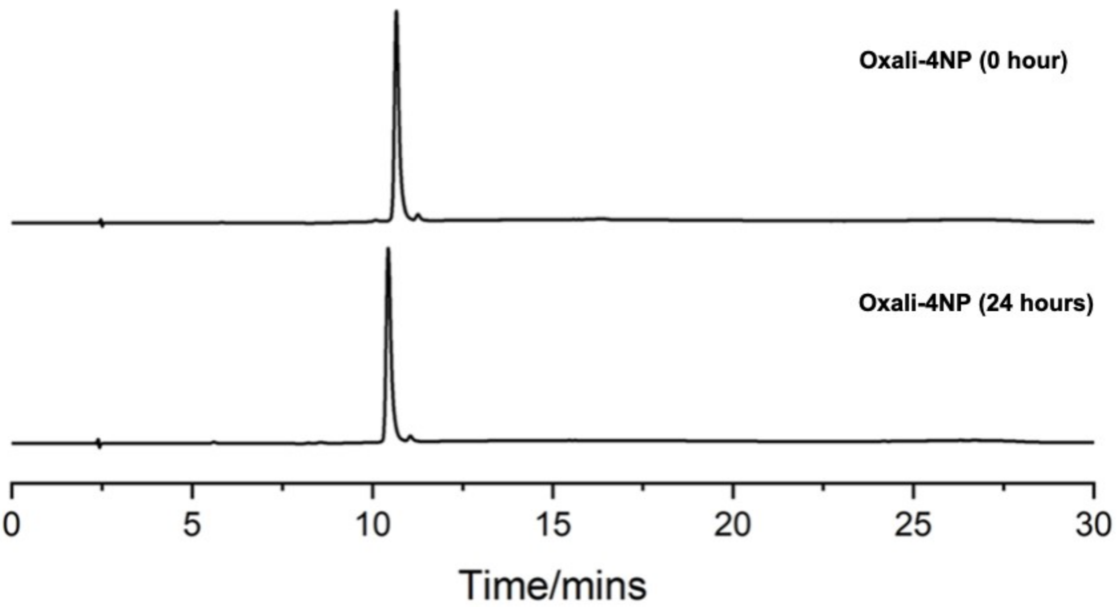

Figure S6. HPLC traces of **Oxali-4NP** (50 µM) in water (with 1% DMF) at 0 and 24 hours (absorption at 340 nm).

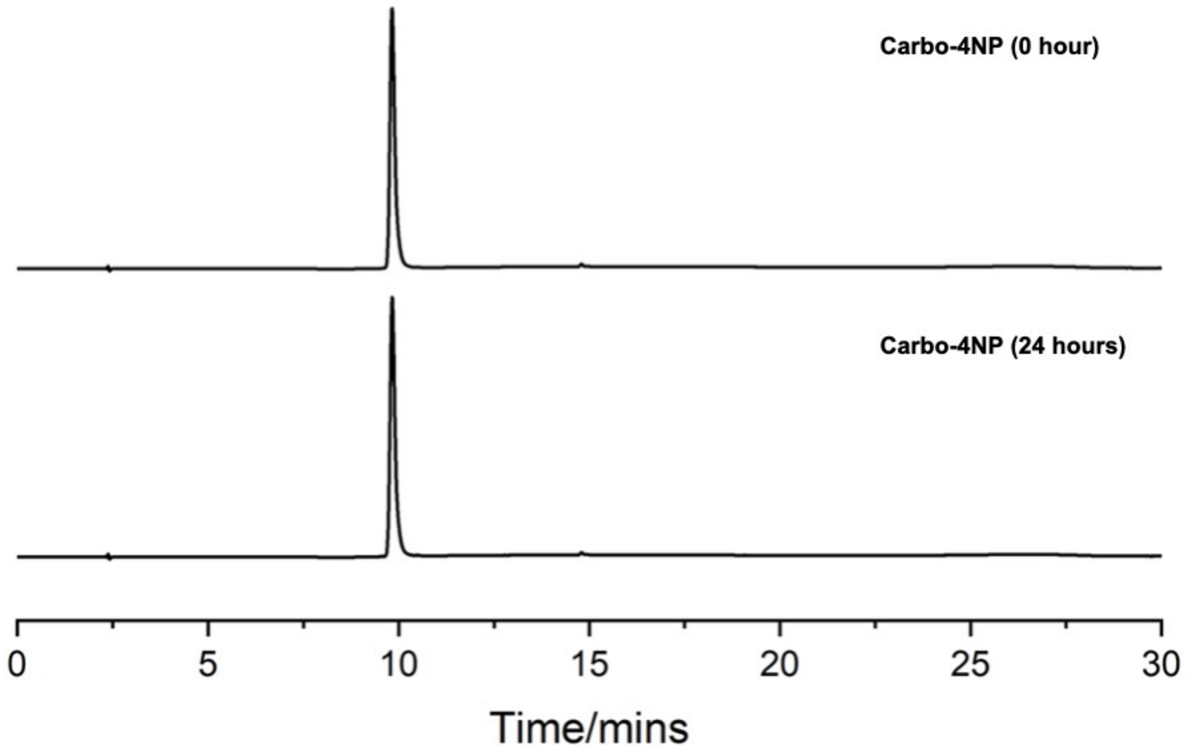

Figure S7. HPLC traces of **Carbo-4NP** (50 µM) in water (with 1% DMF) at 0 and 24 hours (absorption at 340 nm).

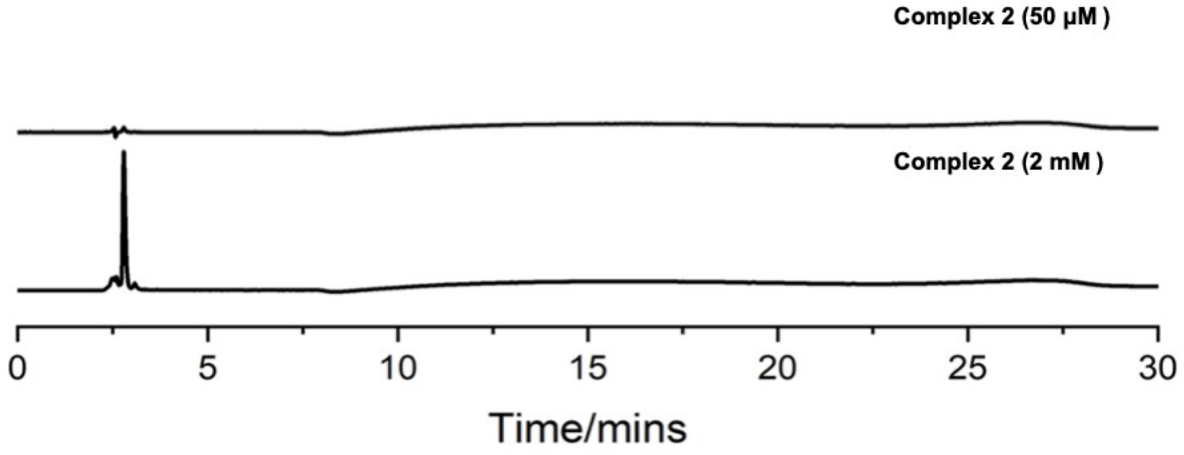

Figure S8. HPLC traces of **Complex 2** in water at different concentrations 50 µM and 2 mM (absorption at 340 nm).

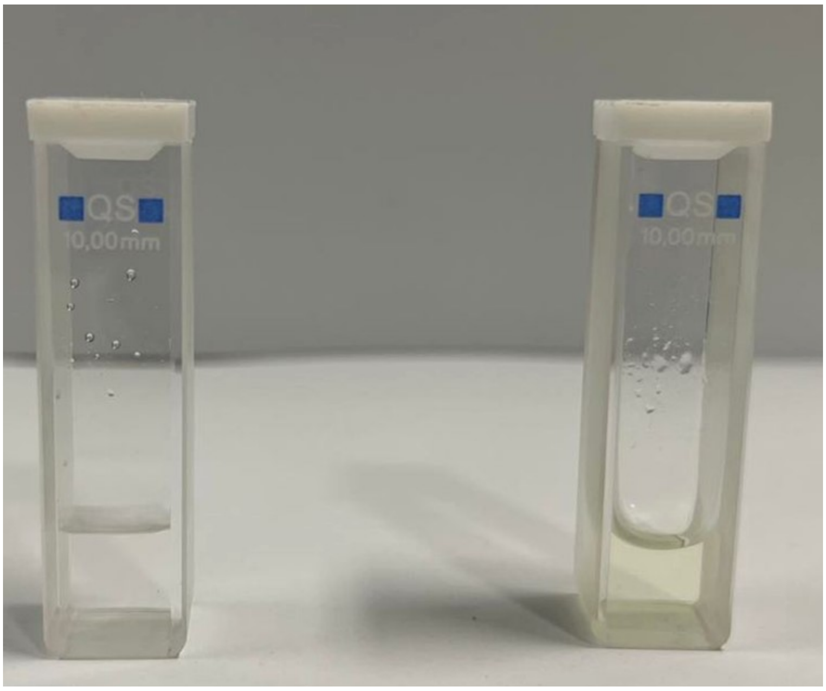

Figure S9. Photograph of solutions of **Cis-4NP** with and without NaAsc. **Left cuvette (Control)**: **Cis-4NP** (50 µM) in PBS buffer (pH 7.40, with 1% DMF) after 7 hours. **Right cuvette**: **Cis-4NP** (50 µM) in PBS buffer (pH 7.40, with 1% DMF) containing 100 equiv. NaAsc (5 mM) after 7 hours incubation.

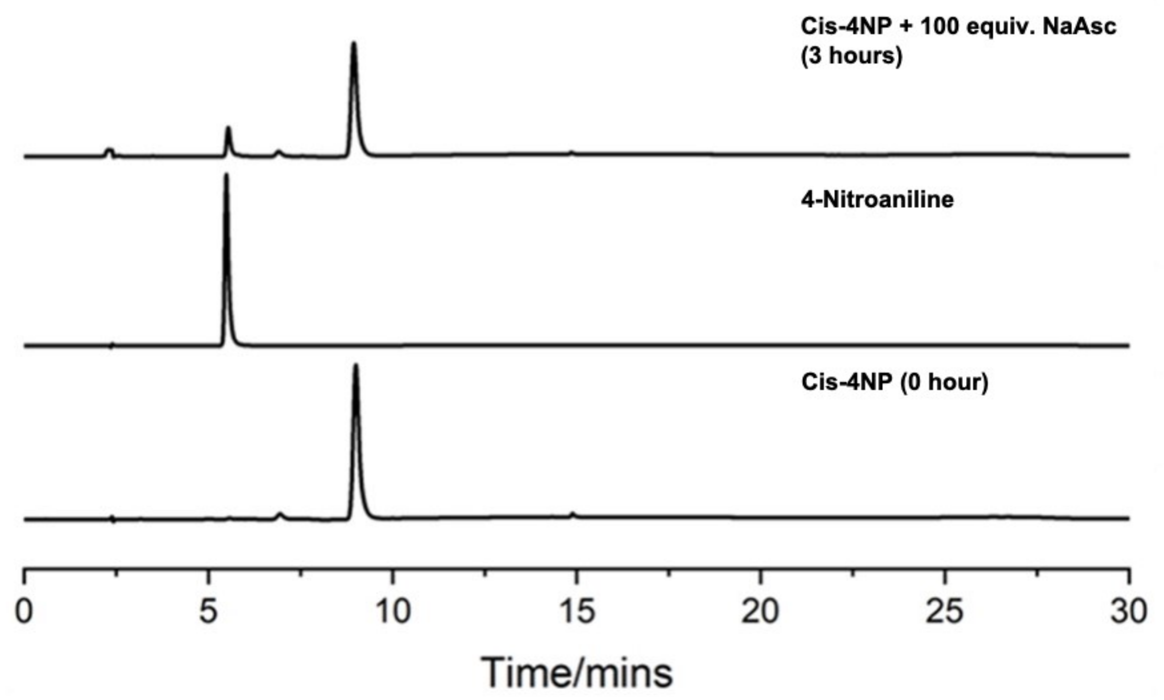

Figure S10. HPLC traces of **(Top) Cis-4NP** (50 µM) with 100 equiv. of NaAsc (5 mM) at 3 hours; **(Middle) 4-nitroaniline** (50 µM) and **(Bottom) Cis-4NP** (50 µM) in water (with 1% DMF) absorption at 340 nm).

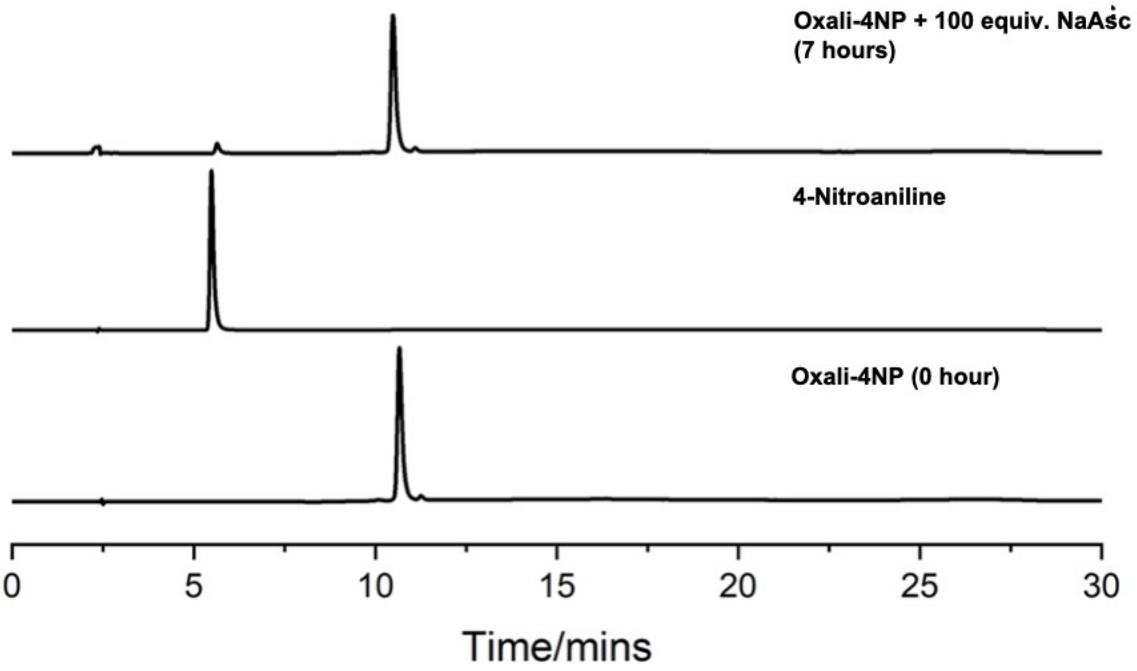

Figure S11. HPLC traces of **(Top) Oxali-4NP** (50 µM) with 100 equiv. NaAsc (5mM) at 7 hours; **(Middle) 4-nitroaniline** (50 µM) and **(Bottom) Oxali-4NP** (50 µM) in water (with 1% DMF) (absorption at 340 nm).

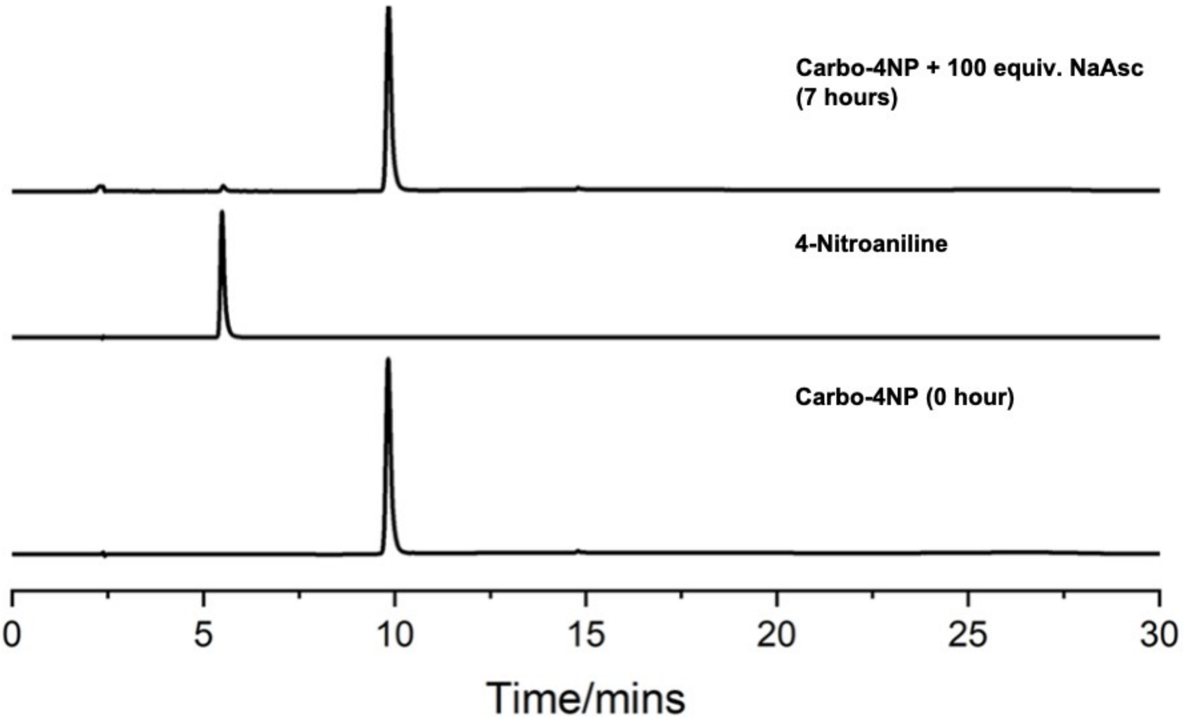

Figure S12. HPLC traces of **(Top) Carbo-4NP** (50 µM) with 100 equiv. NaAsc (5 mM) at 7 hours; **(Middle) 4-Nitroaniline** (50 µM) and **(Bottom) Carbo-4NP** (50 µM) in water (with 1% DMF) (absorption at 340 nm).

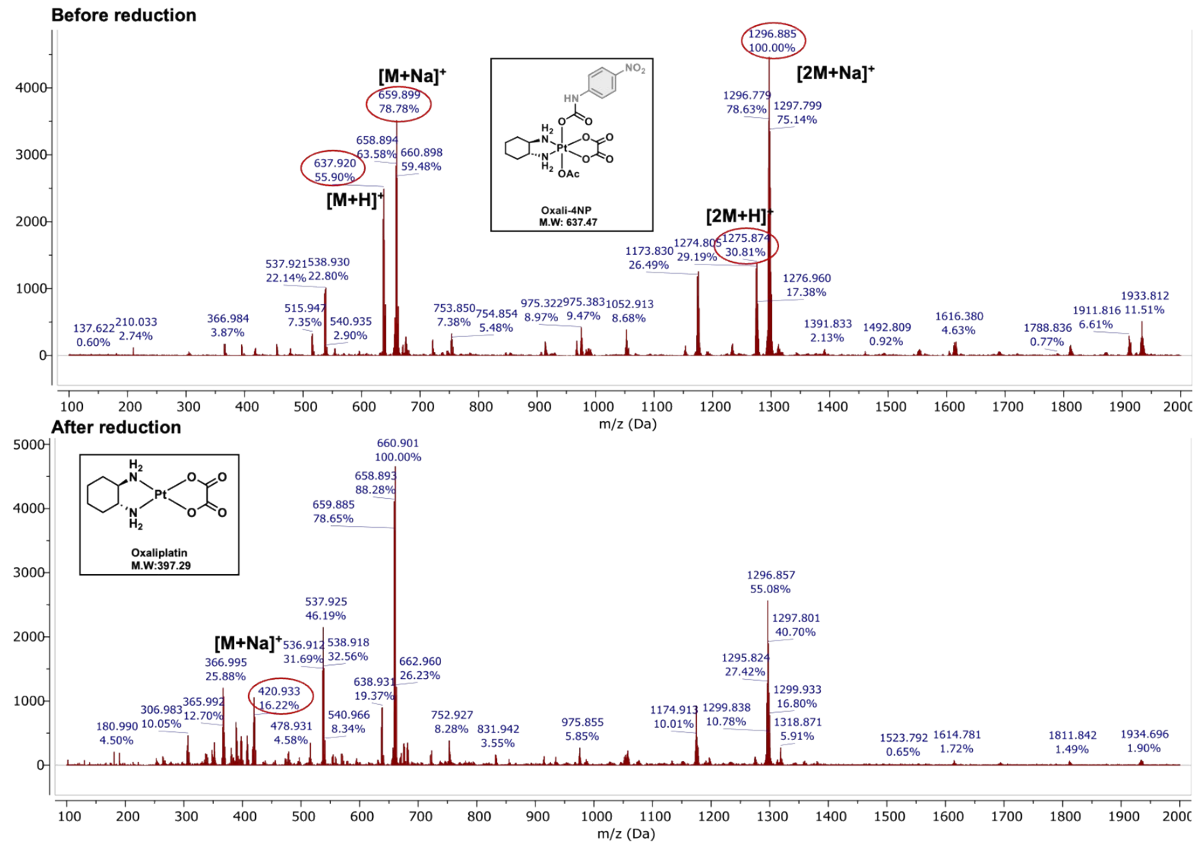

Figure S13. Low-resolution mass spectra of **Oxali-4NP** before and after reduction study.

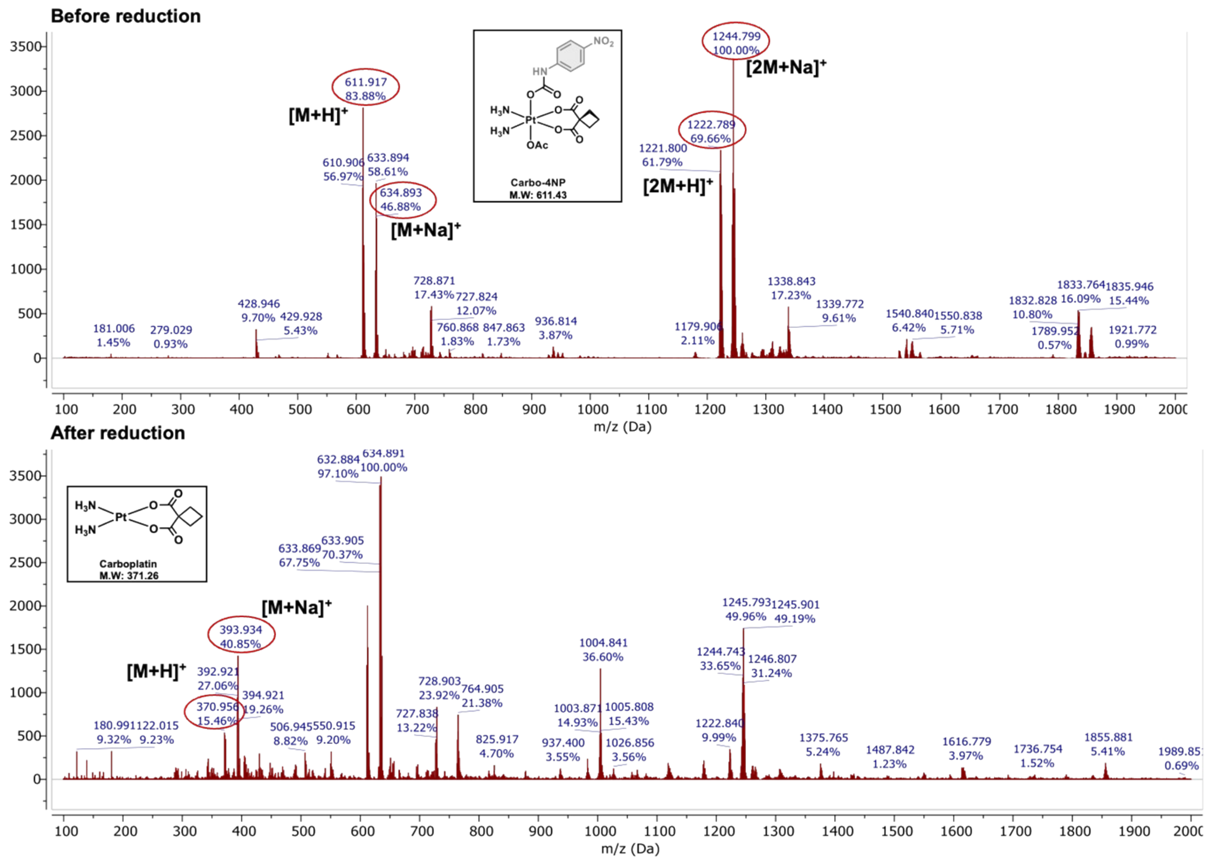

Figure S14. Low-resolution mass spectra of **Carbo-4NP** before and after reduction study.

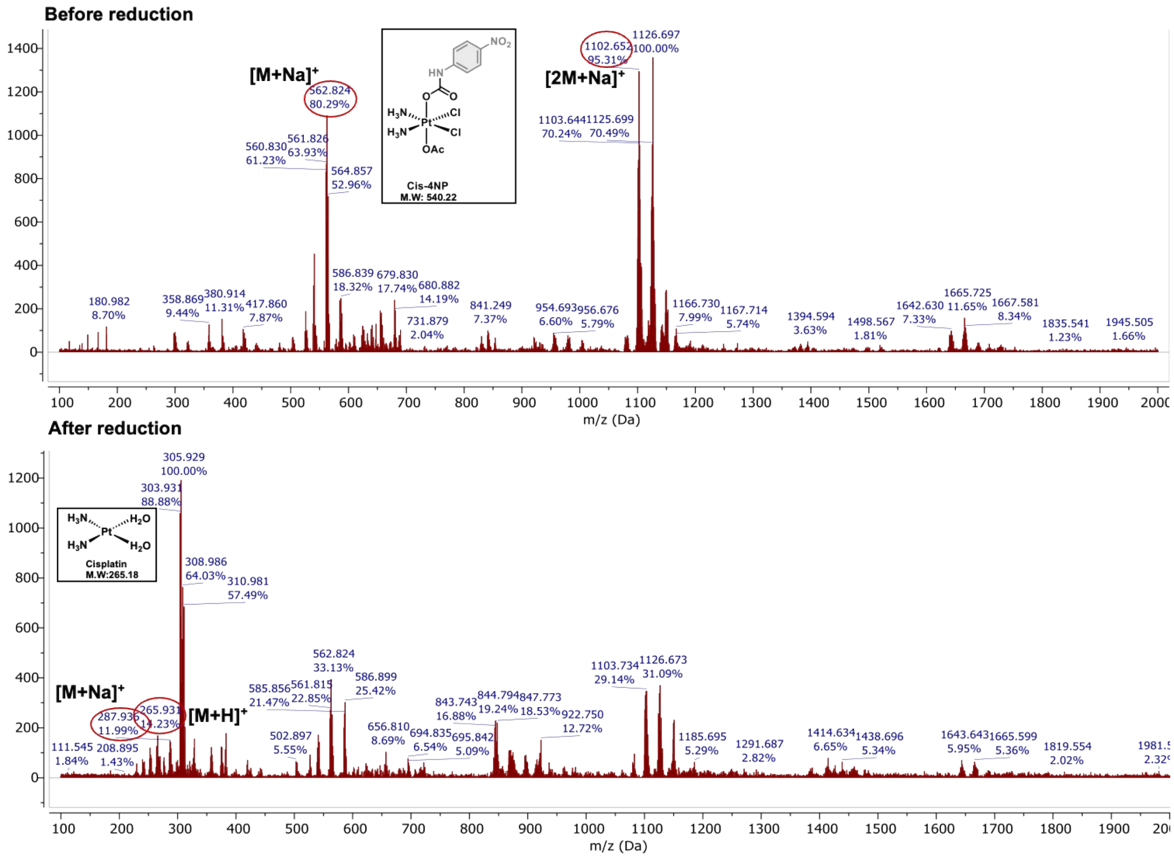

Figure S15. Low-resolution mass spectra of **Cis-4NP** before and after reduction study.

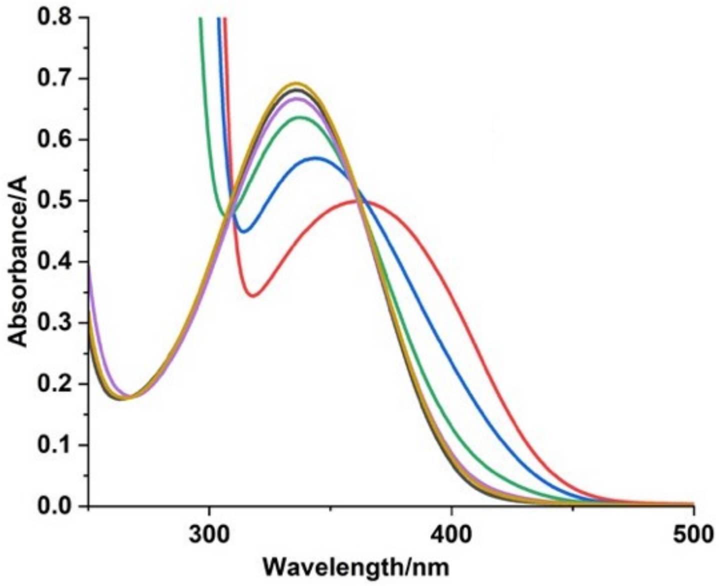

Figure S16. Absorption spectra of **Cis-4NP** (50 µM) in PBS buffer (pH 7.40, with 1% DMF) treated with different reductants and measured after 3 hours. **Red** – 100 equiv. NaAsc. (5 mM), **Blue** – 50 equiv. NaAsc (2.5 mM), **Green** – 10 equiv. NaAsc (0.5 mM), **Purple** – 10 equiv. L-cysteine (0.5 mM), **Brown** – 10 equiv. Glutathione (0.5mM), **Black** – control.

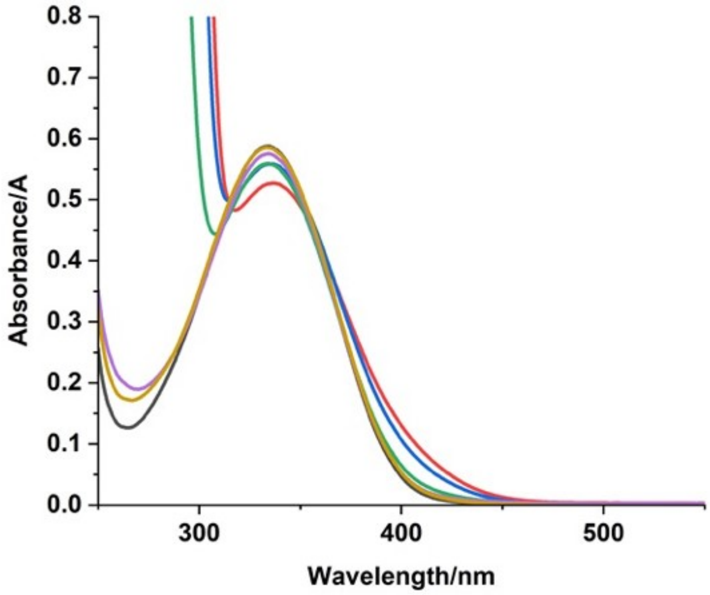

Figure S17. Absorption spectra of **Oxali-4NP** (50 µM) in PBS buffer (pH 7.40, with 1% DMF) treated with different reductants and measured after 7 hours. **Red** – 100 equiv. NaAsc. (5 mM), **Blue** – 50 equiv. NaAsc (2.5 mM), **Green** – 10 equiv. NaAsc (0.5 mM), **Purple** – 10 equiv. L-cysteine (0.5 mM), **Brown** – 10 equiv. Glutathione (0.5mM), **Black** – control.

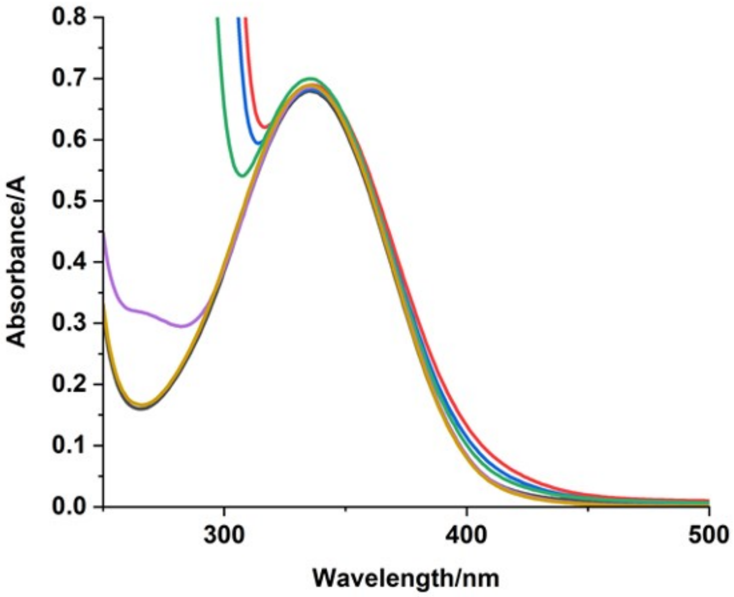

Figure S18. Absorption spectra of **Carbo-4NP** (50 µM) in PBS buffer (pH 7.40, with 1% DMF) treated with different reductants and measured after 7 hours. **Red** – 100 equiv. NaAsc. (5 mM), **Blue** – 50 equiv. NaAsc (2.5 mM), **Green** – 10 equiv. NaAsc (0.5 mM), **Purple** – 10 equiv. L-cysteine (0.5 mM), **Brown** – 10 equiv. Glutathione (0.5mM), **Black** – control.

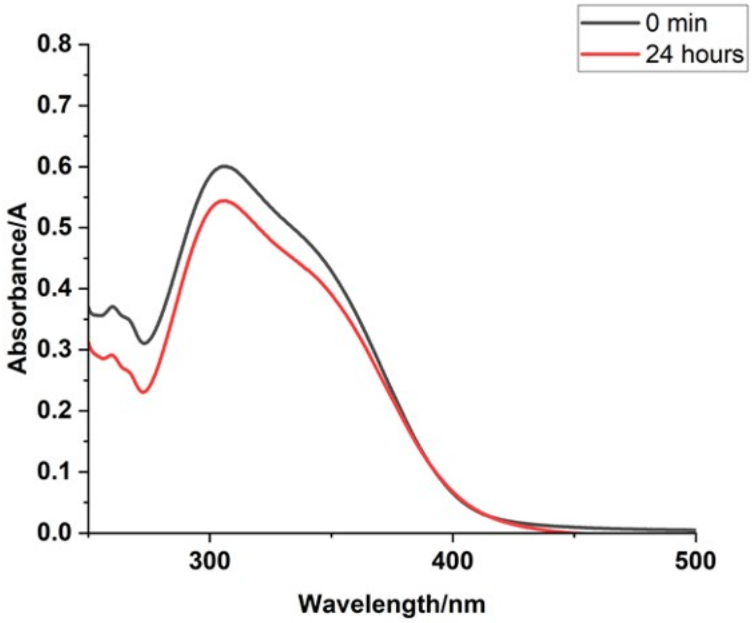

Figure S19. Absorption spectra of **Transazide-4NP** in PBS buffer (pH 7.40, with 1% DMF) at 0 and 24 hours.

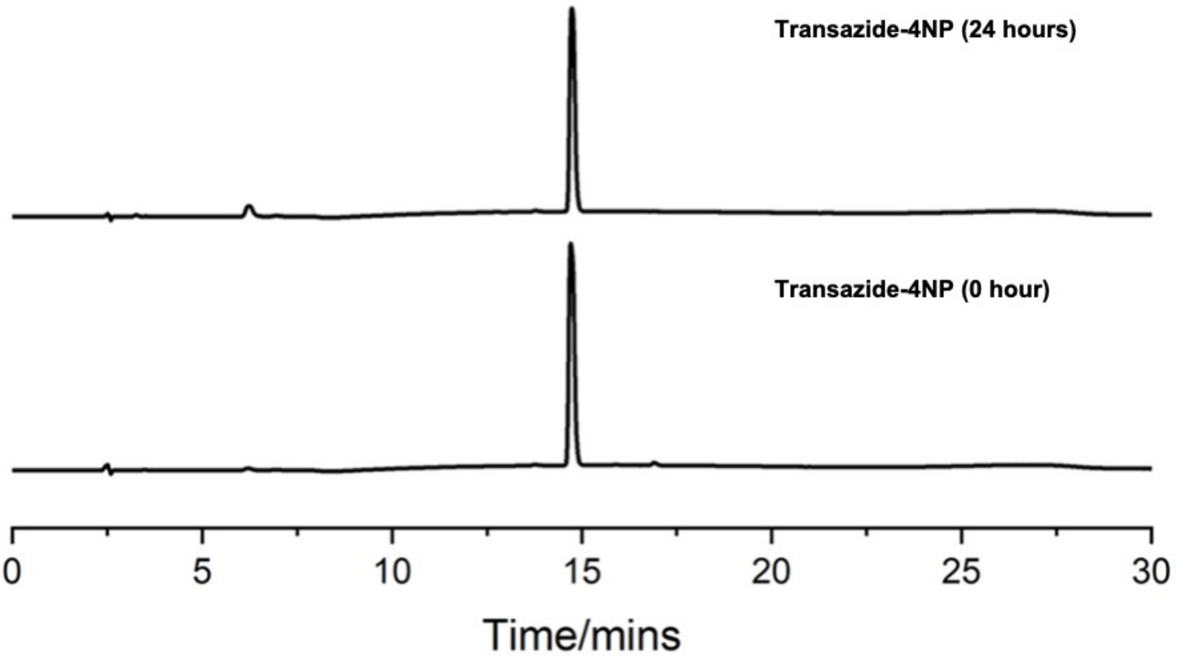

Figure S20. HPLC traces of **Transazide-4NP** (25 µM) in water (with 1% DMF) at 0 and 24 hours (absorption at 340 nm).

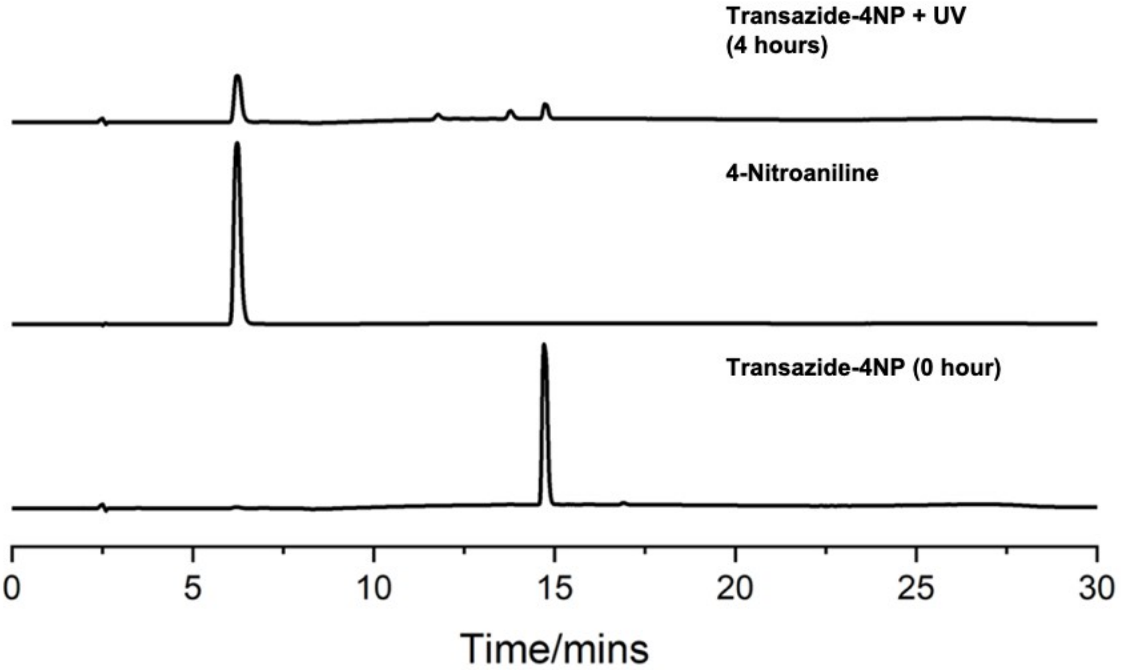

Figure S21. HPLC traces of **Transazide-4NP** (25 µM) in water (with 1% DMF) before and after UV irradiations for 4 hours (absorption at 340 nm).

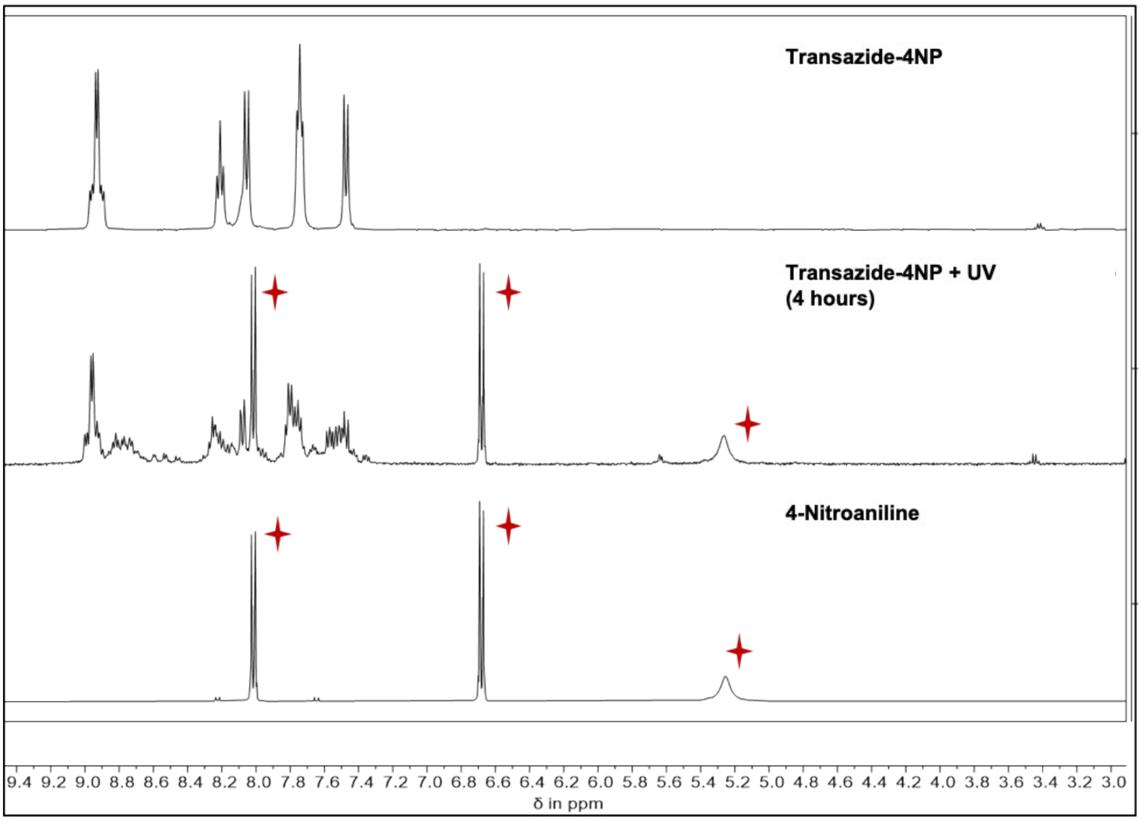

Figure S22. Confirmation of the photomediated release of **4-nitroaniline** from **Transazide-4NP** by ^{1}H-NMR analysis (400 MHz, CD_3CN) **(Top)** ^{1}H-NMR of **Transazide-4NP** **(Middle)** ^{1}H-NMR of **Transazide-4NP** after 4 hours of UV irradiation **(Bottom)** ^{1}H-NMR **of 4-Nitroaniline.**

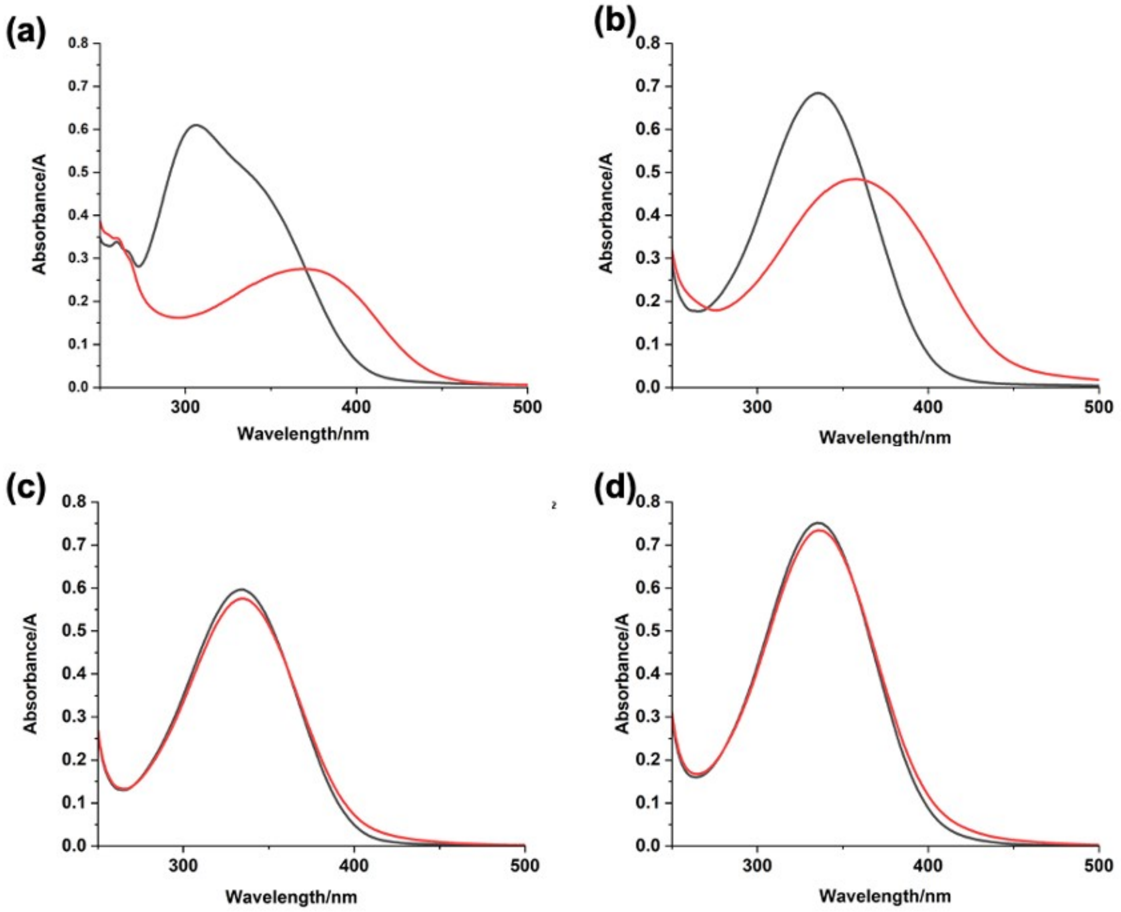

Figure S23. Absorption spectra of **(a) Transazide-4NP** (25 µM) **(b) Cis-4NP** (50 µM) **(c) Oxali-4NP** (50 µM) and **(d) Carbo-4NP** (50 µM) in PBS buffer (pH 7.40, with 1% DMF) irradiated with light for 3 hours.

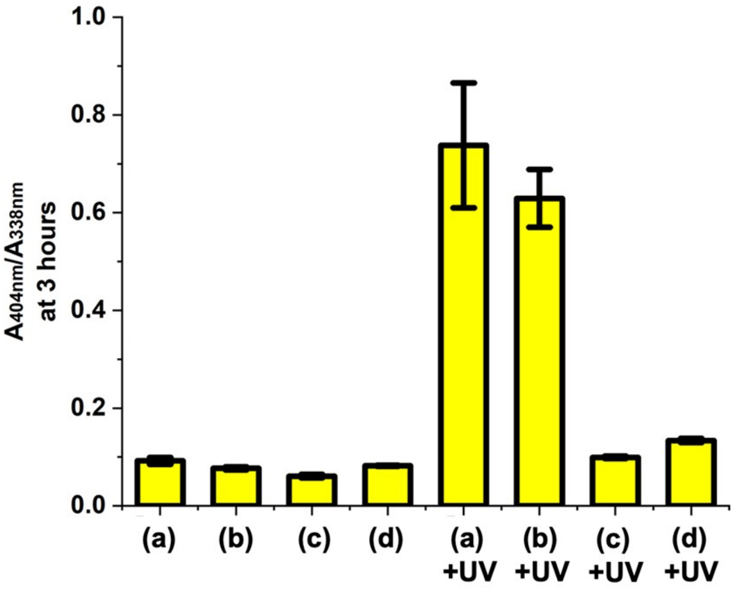

Figure S24. Bar chart of changes in absorption (A404nm/A338nm) after light irradiation (3 hours) of **(a) Transazide-4NP** (25 µM); **(b) Cis-4NP** (50 µM); **(c) Oxali-4NP** (50 µM); and **(d) Carbo-4NP** (50 µM); in PBS buffer (pH 7.40, with 1 % DMF).

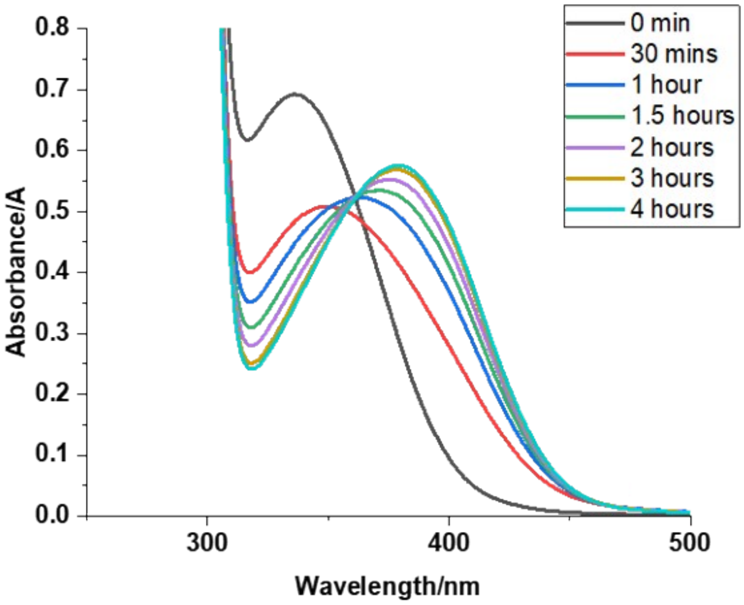

Figure S25. Absorption spectra of **Cis-4NP** (50 µM) in PBS buffer (pH 7.40, with 1 % DMF) containing 100 equiv. NaAsc (5 mM) that was irradiated with UV light over the course of 4 hours.

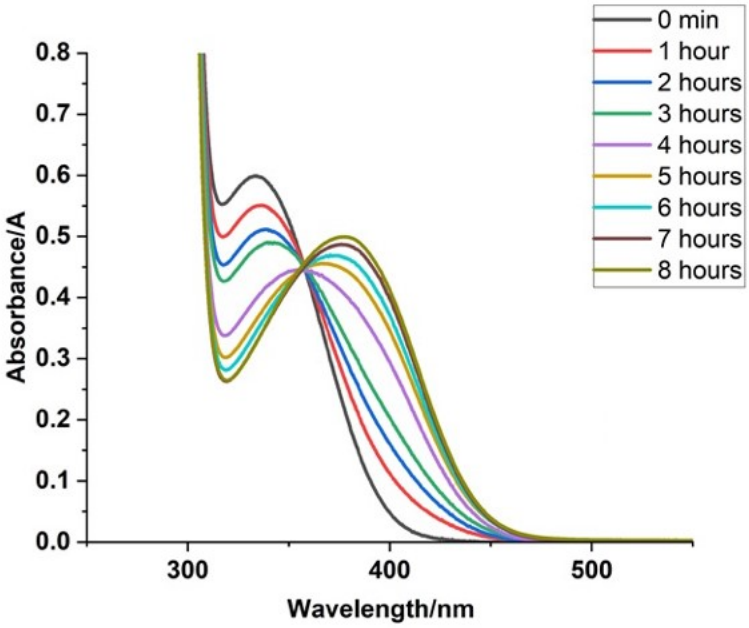

Figure S26. Absorption spectra of **Oxali-4NP** (50 µM) in PBS buffer (pH 7.40, with 1 % DMF) containing 100 equiv. NaAsc (5 mM) that was irradiated with UV light over the course of 8 hours.

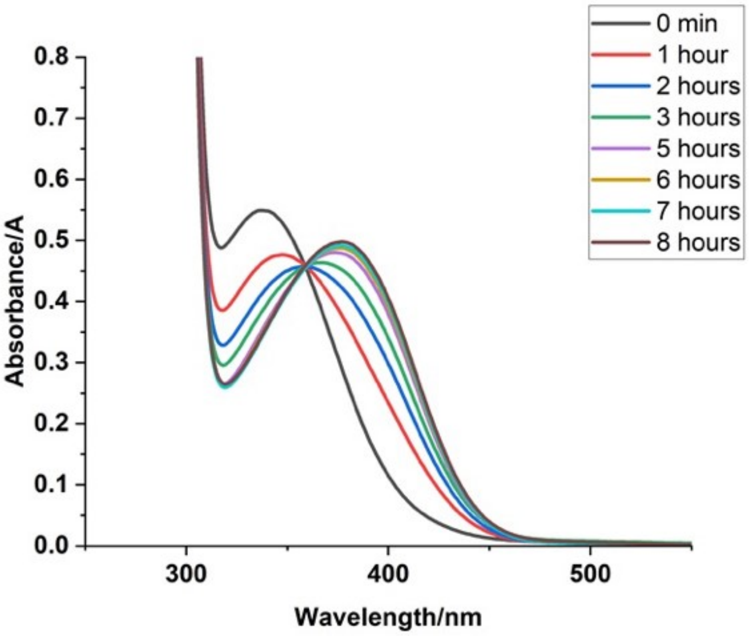

Figure S27. Absorption spectra of **Carbo-4NP** (50 µM) in PBS buffer (pH 7.40, with 1 % DMF) containing 100 equiv. NaAsc (5 mM) that was irradiated with UV light over the course of 8 hours.

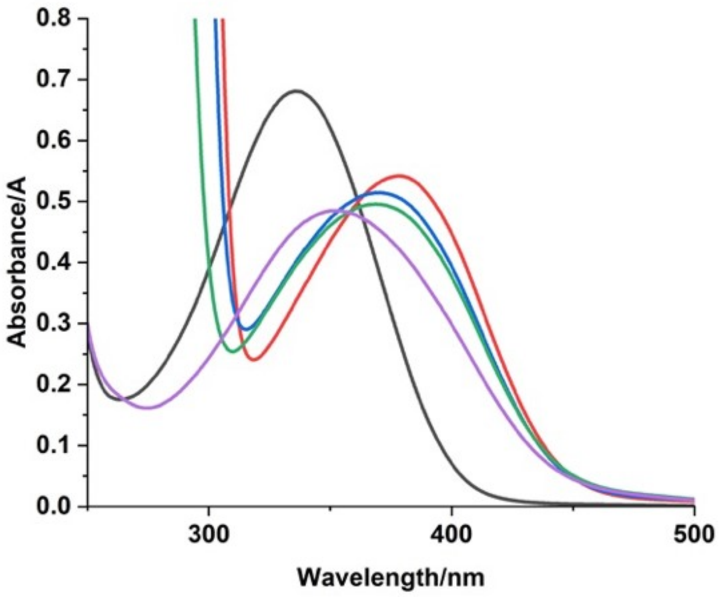

Figure S28. Absorbance spectra of **Cis-4NP** (50 µM) under different photoreduction conditions in PBS buffer (pH 7.40, with 1% DMF) at 3 hours **Red** – UV irradiation with 100 equiv. NaAsc (5 mM); **Blue** – UV irradiation with 50 equiv. NaAsc (2.5 mM); **Green** – UV irradiation with 10 equiv. NaAsc (0.5 mM); **Purple** – UV irradiation; **Black** – control.

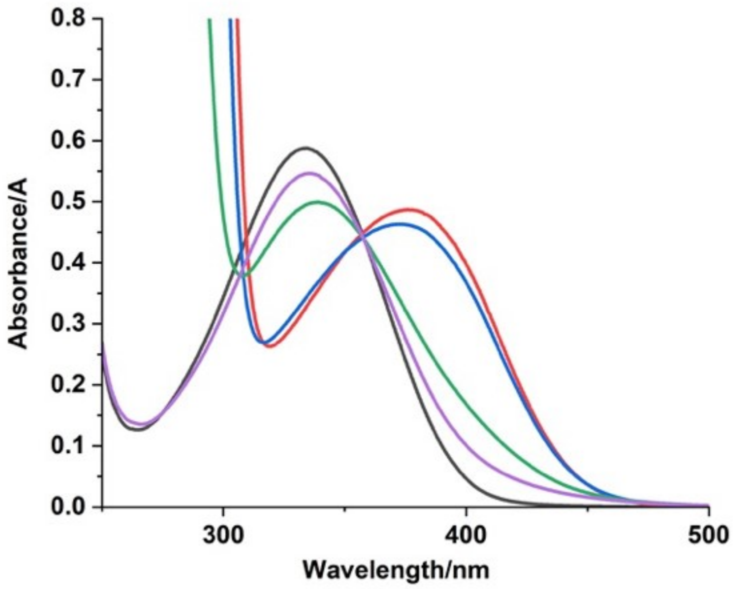

Figure S29. Absorbance spectra of **Oxali-4NP** (50 μM) under different photoreduction conditions in PBS buffer (pH 7.40, with 1% DMF) at 7 hours **Red** – UV irradiation with 100 equiv. NaAsc (5 mM); **Blue** – UV irradiation with 50 equiv. NaAsc (2.5 mM); **Green** – UV irradiation with 10 equiv. NaAsc (0.5 mM); **Purple** – UV irradiation; **Black** – control.

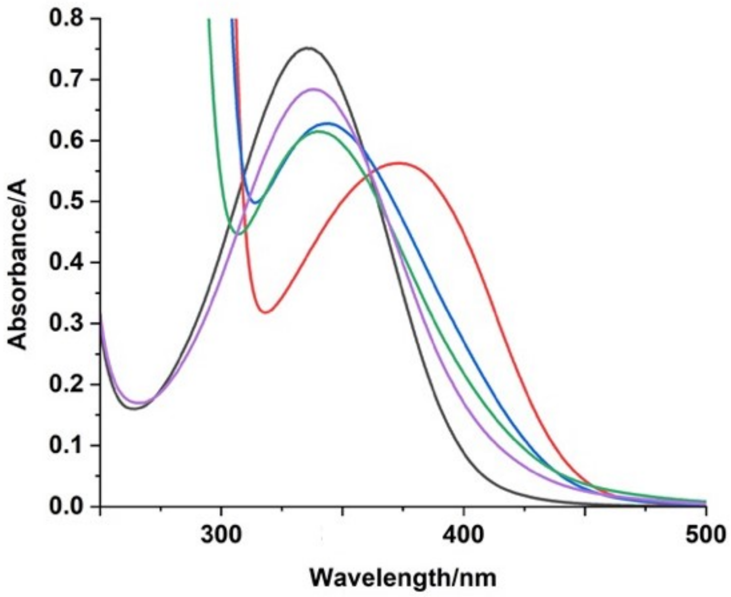

Figure S30. Absorbance spectra of **Carbo-4NP** (50 µM) under different photoreduction conditions in PBS buffer (pH 7.40, with 1% DMF) at 7 hours **Red** – UV irradiation with 100 equiv. NaAsc (5 mM); **Blue** – UV irradiation with 50 equiv. NaAsc (2.5 mM); **Green** – UV irradiation with 10 equiv. NaAsc (0.5 mM); **Purple** – UV irradiation; **Black** – control.

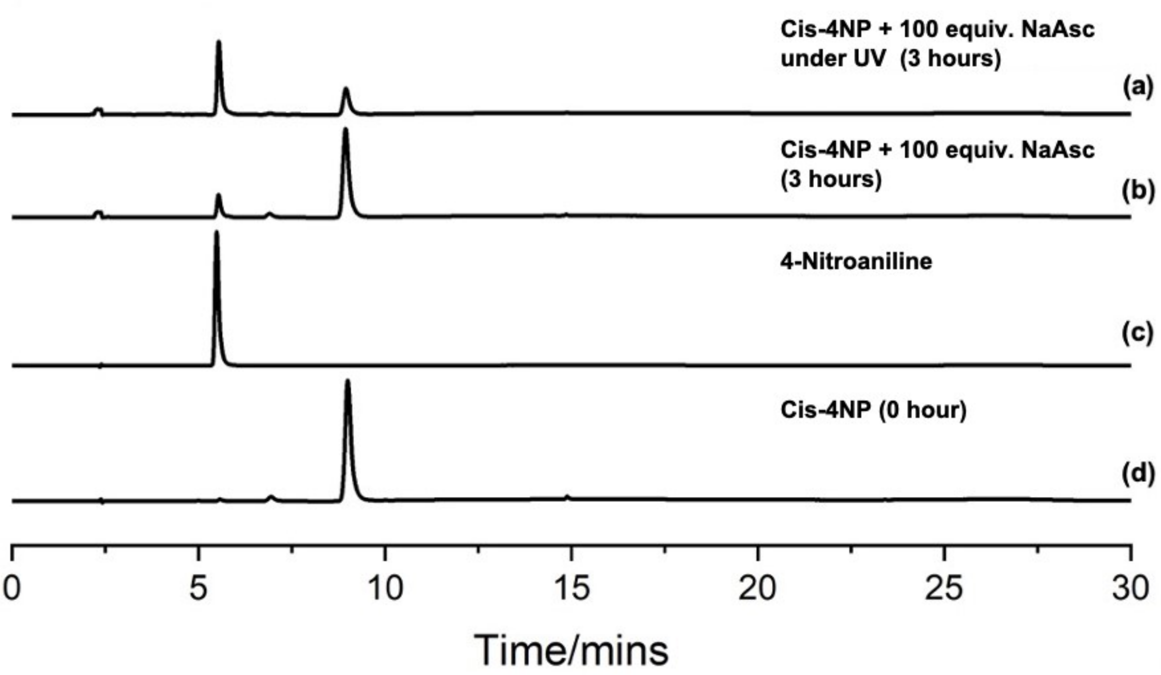

Figure S31. HPLC traces (Absorption at 340 nm) of **(a) Cis-4NP** (50 μM) with 100 equiv. NaAsc (5 mM) under UV irradiations at 3 hours; **(b) Cis-4NP** (5 0μM) with 100 equiv. NaAsc (5 mM) at 3 hours; **(c) 4-Nitroaniline** (50 μM); and **(d) Cis-4NP** (50 μM) in water (with 1% DMF).

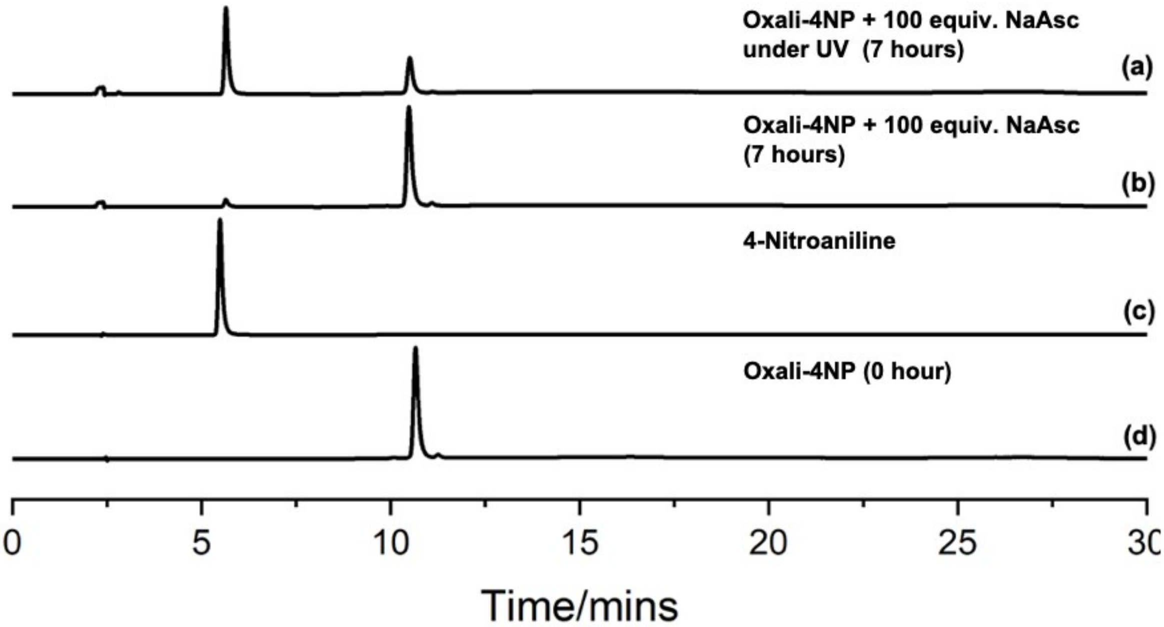

Figure S32. HPLC traces (Absorption at 340 nm) of **(a) Oxali-4NP** (50 µM) with 100 equiv. NaAsc (5 mM) under UV irradiations at 7 hours; **(b) Oxali-4NP** (50 µM) with 100 equiv. NaAsc (5mM) at 7 hours; **(c) 4-Nitroaniline** (50 µM); and **(d) Oxali-4NP** (50 µM) in water (with 1% DMF).

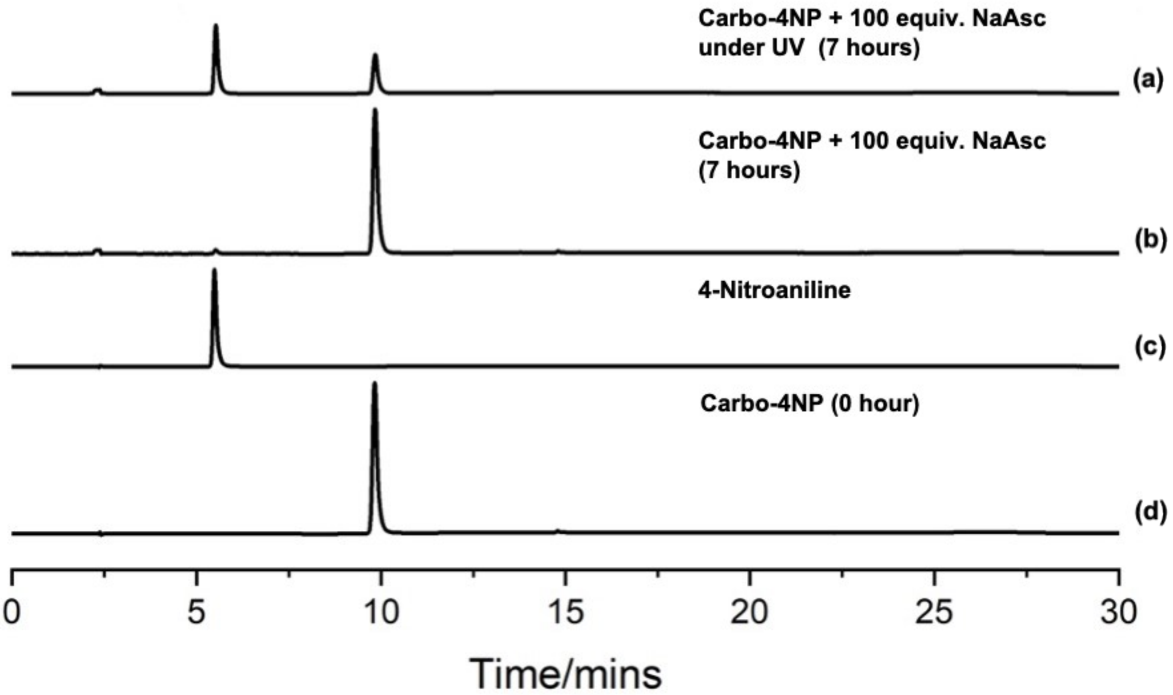

Figure S33. HPLC traces (absorption at 340 nm) of **(a) Carbo-4NP** (50 µM) with 100 equiv. NaAsc (5 mM) under UV irradiations at 7 hours; **(b) Carbo-4NP** (50 µM) with 100 equiv. NaAsc (5 mM) at 7 hours; **(c) 4-Nitroaniline** (50 µM); and **(d) Carbo-4NP** (50 µM) in water (with 1% DMF).

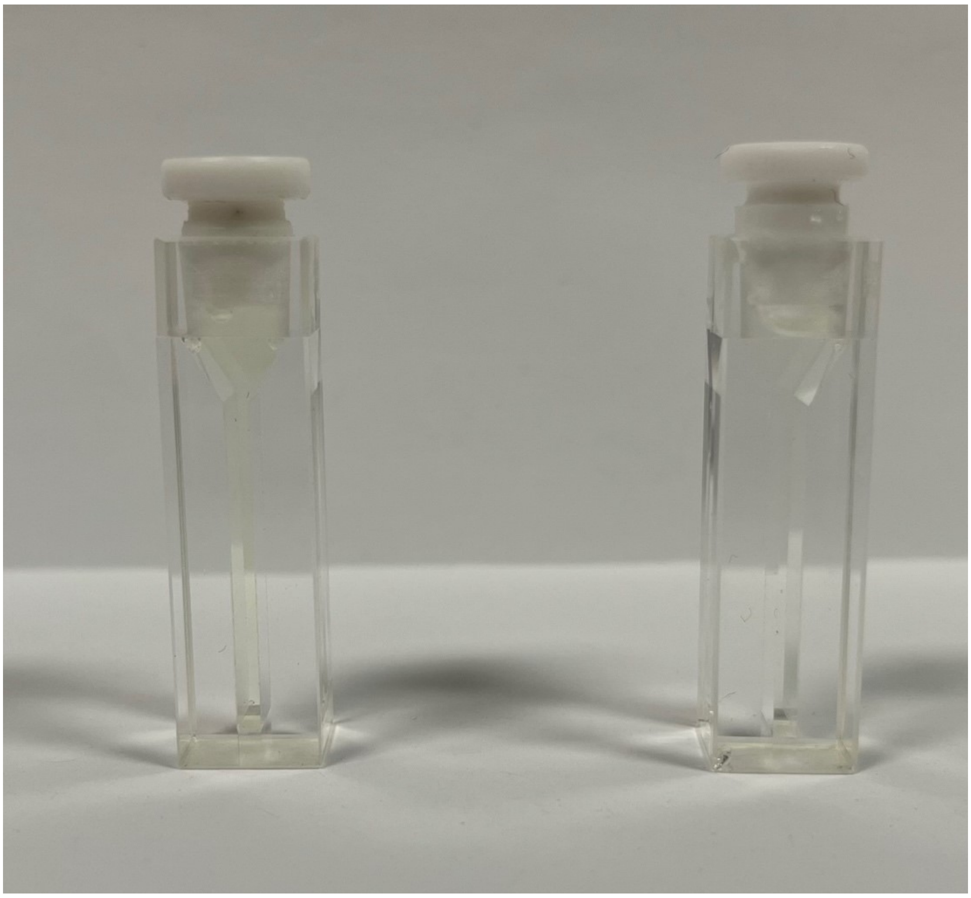

Figure S34. Photograph of solutions of **Transazide-4NP** with and without NaAsc. **Left Cuvette: Transazide-4NP** (25 µM) in PBS buffer (pH 7.40, with 1% DMF) containing 100 equiv. NaAsc (2.5 mM) after 24 hours incubation. **Right Cuvette: (Control) Transazide-4NP** (25 µM) in PBS buffer (pH 7.40, with 1% DMF) after 24 hours incubation.

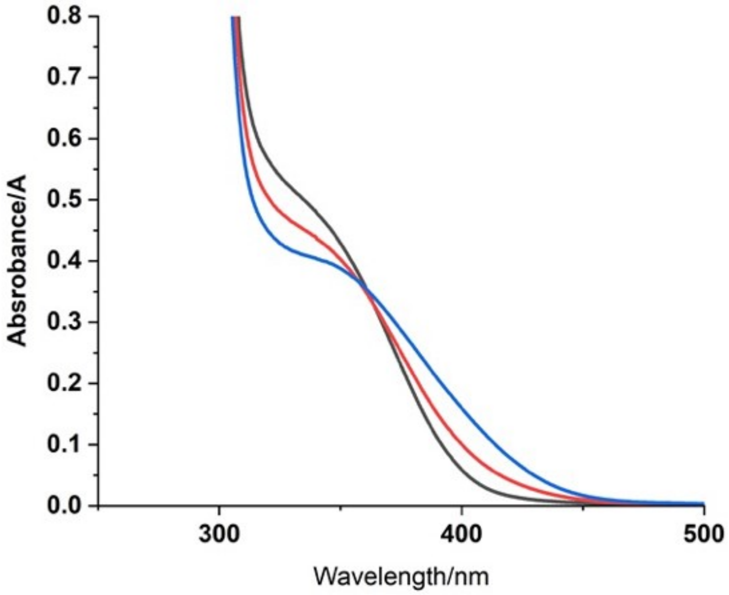

Figure S35. Absorbance spectra of **Transazide-4NP** (25 µM) in PBS buffer (pH 7.40, with 1% DMF) containing 100 equiv. NaAsc (2.5 mM) at 0 hour **(black)**; 7 hours **(red)** and 24 hours **(blue)**.

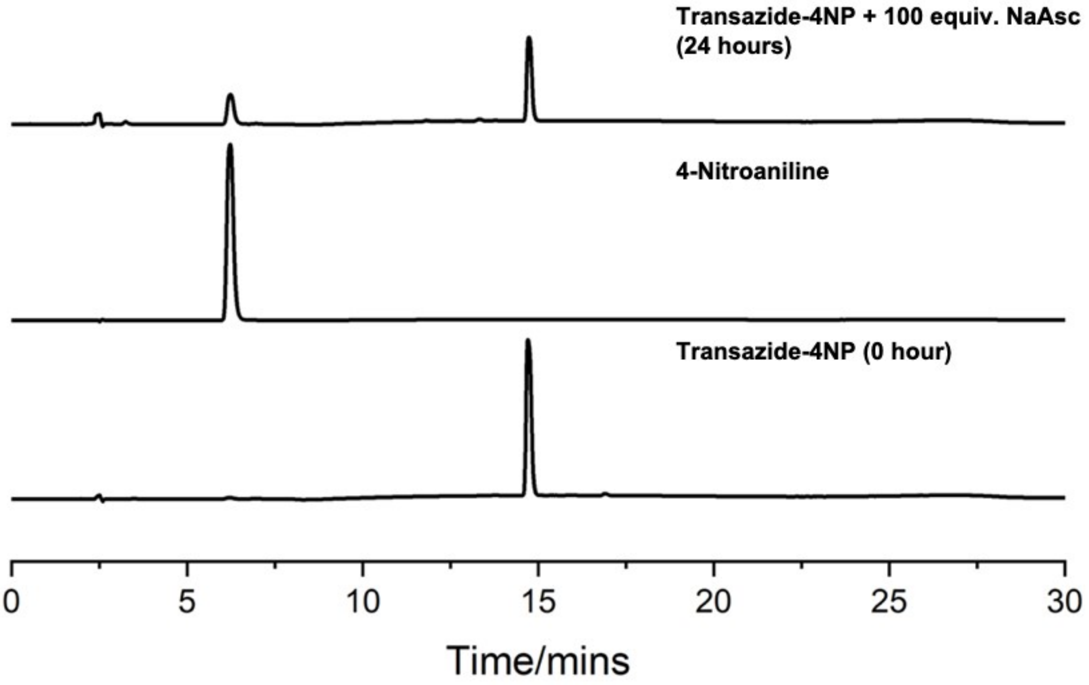

Figure S36. HPLC traces of **(Top) Transazide-4NP** (25 µM) with NaAsc (100 equiv., 5 mM) at 24 hours; **(Middle) 4-Nitroaniline** (50 µM) and **(Bottom) Transazide-4NP** (25 µM) in water (with 1% DMF).

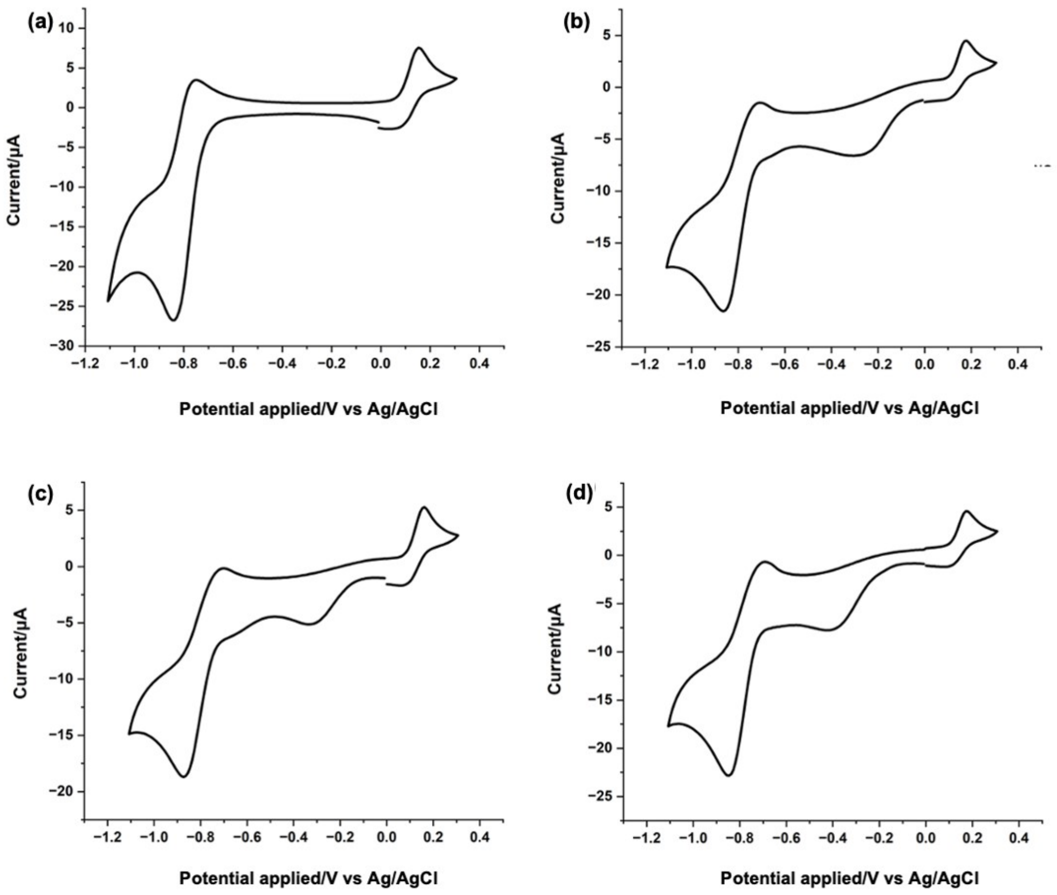

Figure S37. Cyclic Voltammogram of a 3mm glassy carbon electrode immersed in 1mM solution of **(a) 4-nitroaniline; (b) Cis-4NP; (c) Oxali-4NP** and **(d) Carbo-4NP** in PBS buffer (pH 7.40, with 10 % DMF) at a scan rate of 0.1 V/s.

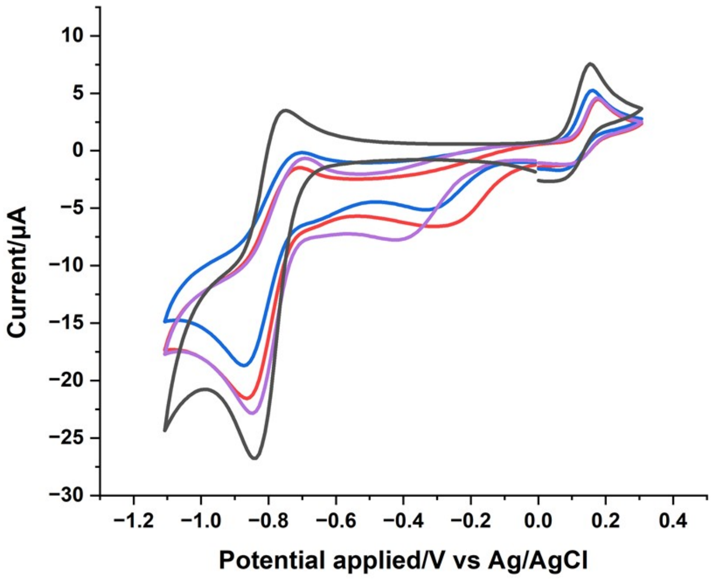

Figure S38. Overlay of Cyclic Voltammogram of a 3mm glassy carbon electrode immersed in 1mM solution of **4-nitroaniline (Black); Cis-4NP (Red); Oxali-4NP (Blue) and Carbo-4NP (Purple)** in PBS buffer (pH 7.40, with 10% DMF) at a scan rate of 0.1V/s.

4. Synthetic Procedures

General procedure for Complex 1-3

Complexes 1-3 were synthesized according to modified literature reported procedure.[1-4] Pt(II) precursors (100 mg) was suspended in acetic acid with 50% aq. H_2O_2. The reaction mixture was left to stir in the dark at room temperature until a clear solution was obtained. The solvent was concentrated under reduced pressure and diethyl ether was added to the resulting solution. The formed precipitate was collected by centrifugation and washed with diethyl ether, acetone, and dichloromethane. No further purification was carried out as the purity of each isolated complex was deemed suitable for the subsequent synthetic step.

[Pt(NH$_3$)$_2$Cl$_2$(OH)(OAc)] – Complex 1

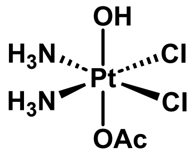

Yield (98 mg, 0.26 mmol, 78%).^{1}H NMR (400 MHz, DMSO-d$_6$) δ 6.07-5.81 (m, 6H), 1.86 (t, 3H). ^{1}H NMR spectrum data was consistent with a literature report. [1]

[Pt(DACH)(ox)(OH)(Oac)] Complex 2

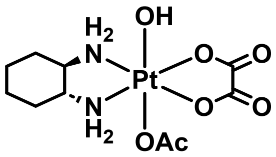

Yield (93 mg, 0.20 mmol, 79%).^{1}H NMR (400 MHz, DMSO-d$_6$) δ 8.57 (br, s, 1H), 8.16 (br, s, 1H), 7.79 (br, s, 1H), 7.10 (br, s, 1H), 2.53 (obscured by DMSO-d$_6$ signal, 2H), 2.06 (m, 2H), 1.90 (s, 3H), 1.54-1.28 (m, 4H), 1.11 (m, 2H). ^{1}H NMR spectrum data is consistent with a literature report. [2]

[Pt(NH$_3$)$_2$(CBDCA)(OH)(Oac)] Complex 3

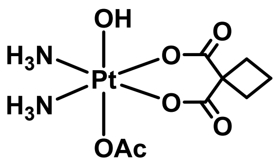

Yield (99 mg, 0.22 mmol, 83 %).^{1}H NMR (400 MHz, DMSO-d$_6$) 6.02-5.76 (m, 6H), 2.53 (signal obscured by DMSO-d$_6$ signal, 2H), 2.48 (signal obscured by DMSO-d$_6$ signal, 2H), 1.86 (s, 3H), 1.78 (m, 2H). ^{1}H NMR spectrum data is consistent with a literature report.[3]

***t,t,t*-[Pt(N$_3$)$_2$(OH)$_2$(py)$_2$] Complex 4**

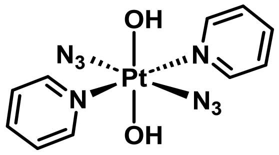

t,t,t-[Pt(N$_3$)$_2$ (py)$_2$][4] (100mg) was suspended in 34% aq. H_2O_2 (8 ml) and stirred at 45 ^{o}C for 3 hours. The resulting yellow solution was then concentrated under reduced pressure. To the remaining solution, ethanol and diethyl ether were added and left to crystalise at -4 ^{o}C overnight. Yellow crystals were collected by filtration and washed with ice cold ethanol and ether. Yield (89 mg, 0.23 mmol, 82 %) ^{1}H NMR (400 MHz, D_2O) 8.80 (dd, J = 27 Hz, 4H), 8.29 (t, J =7 Hz, 2H), 7.83 (t, J = 7 Hz, 4H). ^{1}H NMR spectrum data is consistent with a literature report.[4]

General procedure for Cis-4NP, Oxali-4NP, Carbo-4NP and Transazide-4NP

4-nitrophenyl isocyanate (4 equiv.) was added to a (suspension/solution) of Pt(IV) complex (**1**-**4**) (1 equiv.) in DMF. The reaction mixture was left to stir at room temperature in the dark for approximately 3 hours and subsequently purified via silica chromatography (100% hexane to 100% EtOAc to CH_2Cl_2/MeOH (90:10). The solvent was removed under reduced pressure and the resulting solid was further lyophilised to afford the final 4-NP Pt(IV) complex as yellow solid.

[Pt(NH_3)$_2$$Cl_2$($OOCNHC_6H_4NO_2$)(OAc)] Cis-4NP

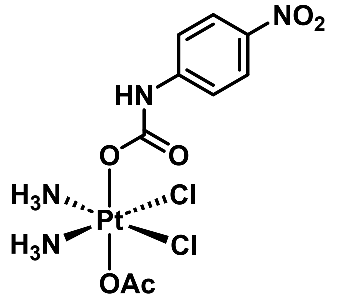

Yellow solid. Yield (31 mg, 0.057 mmol, 30%). ^{1}H NMR (400 MHz, CD_3CN) δ 8.11 (d, *J* = 8 Hz, 2H), 7.58 (d, *J* = 8 Hz, 2H), 5.99 (m, 6H), 2.02 (s, 3H). ^{13}C NMR (400 MHz, CD_3CN) δ 180.93, 161.51, 147.29, 142.95, 125.87, 22.65. ^{195}Pt NMR (500 MHz, DMSO-d_6) δ 1254.51. HRMS: m/z calculated for $C_9H_{15}Cl_2N_4O_6Pt$: 540.0011 for $[M+H]^+$, found 540.0020.

[Pt(DACH)(ox)(OOCNHC$_6$H$_4$NO$_2$)(OAc)] Oxali-4NP

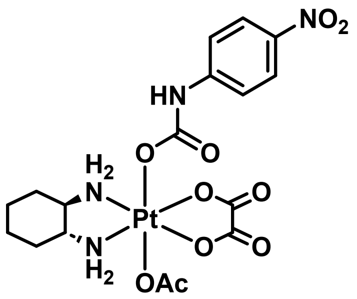

Pale yellow solid. Yield (42 mg, 0.066 mmol, 44%).^{1}H NMR (400 MHz, CD$_3$CN) δ 8.26 (s, 1H), 8.12 (d, *J* = 8 Hz, 2H), 7.55 (d, *J* = 8 Hz, 2H), 2.95 (td, *J* = 9.4, 3.4 Hz, 1H), 2.82 (td, *J* = 9.4, 3.4 Hz, 1H), 2.26 (t, *J* = 9.1 Hz, 2H),2.02 (s, 3H), 1.66-1.44 (m, 4H), 1.27 (m, *J* = Hz, 2H). ^{13}C NMR (400 MHz, CD$_3$CN) δ 181.42, 164.17, 161.65, 146.86, 143.17, 125.88, 62.76, 62.60. ^{195}Pt NMR (500 MHz, DMSO-d$_6$) δ 1632.40. HRMS: m/z calculated for $C_{17}H_{23}N_4O_{10}Pt$: 638.1057 for $[M+H]^+$, found 638.1055.

[Pt(NH$_3$)$_2$(CBDCA)(OOCNHC$_6$H$_4$NO$_2$)(OAc)] Carbo-4NP

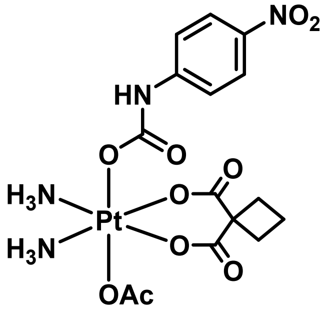

Yellow solid. Yield (45 mg, 0.074mmol, 46%). ^{1}H NMR (400 MHz, DMSO-d$_6$) δ 9.82 (s, 1H), 8.10 (d, *J* = 8 Hz, 2H), 7.64 (d, *J* = 8 Hz, 2H), 6.2-6.8 (m, br, 6H), 2.59 (t, *J* = 8 Hz, 2H), 2.44 (d, *J* = 8 Hz, 2H), 1.92 (s, 3H), 1.83 (p, *J* = 8 Hz, 2H). ^{13}C NMR (400 MHz, DMSO-d$_6$) δ 177.43, 176.15, 159.05, 147.37, 140.52, 124.83, 117.16, 55.43, 33.18, 29.11, 22.37, 15.75 ^{195}Pt NMR (500 MHz, DMSO-d$_6$) δ 1971.52. HRMS: m/z calculated for $C_{15}H_{21}N_4O_{10}Pt$: 612.0900 for $[M+H]^+$, found 612.0901.

t,t,t-[Pt(N_3)$_2$(py)$_2$ ($OOCNHC_6H_4NO_2$)OH] - Transazide-4NP

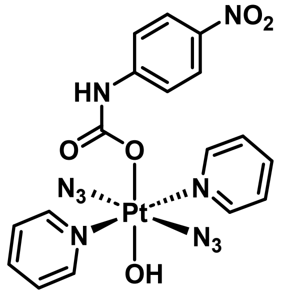

Yellow solid. Yield (54 mg, 0.085mmol, 57%).^{1}H NMR (400 MHz, CD_3CN) δ 8.93 (d, J = 27, 6.5 Hz, 4H), 8.21 (t, J = 8.5 Hz, 2H), 8.06 (d, J = 8.5 Hz, 3H), 7.74 (t, J = 6.5 Hz, 4H), 7.47 (d, J = 8.5 Hz, 2H). ^{13}C NMR (600 MHz, CD_3CN) δ 157.34, 150.50, 148.53, 143.16, 142.24, 127.24, 125.88, 117.60. ^{195}Pt NMR (500 MHz, DMSO-d_6) δ 1008.58. HRMS: m/z calculated for $C_{17}H_{16}N_{10}O_5Pt$: 636.1028 for [M^+], found 636.1026.

5. NMR Spectra

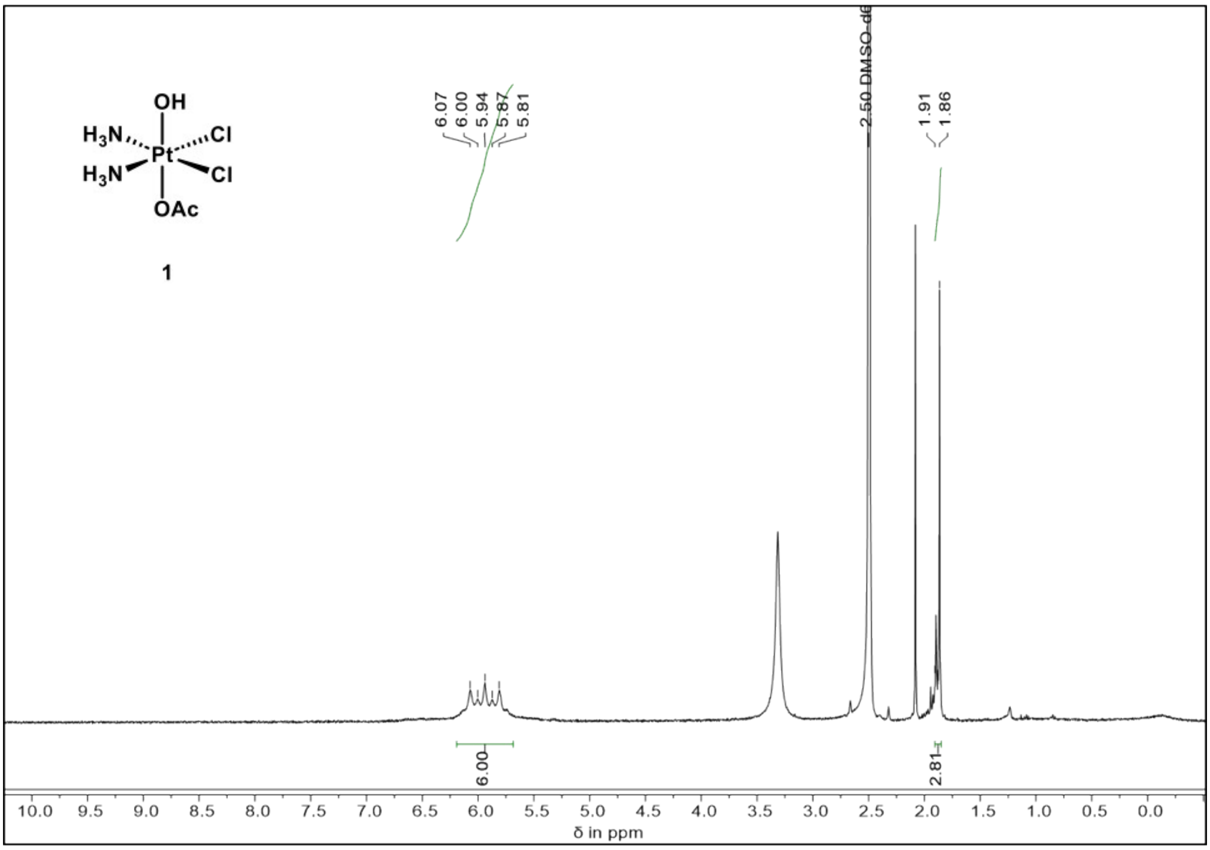

Figure S37. ^{1}H NMR (400 MHz, DMSO-d_6) of **Complex 1 [Pt(NH$_3$)$_2$Cl$_2$(OH)(OAc)].**

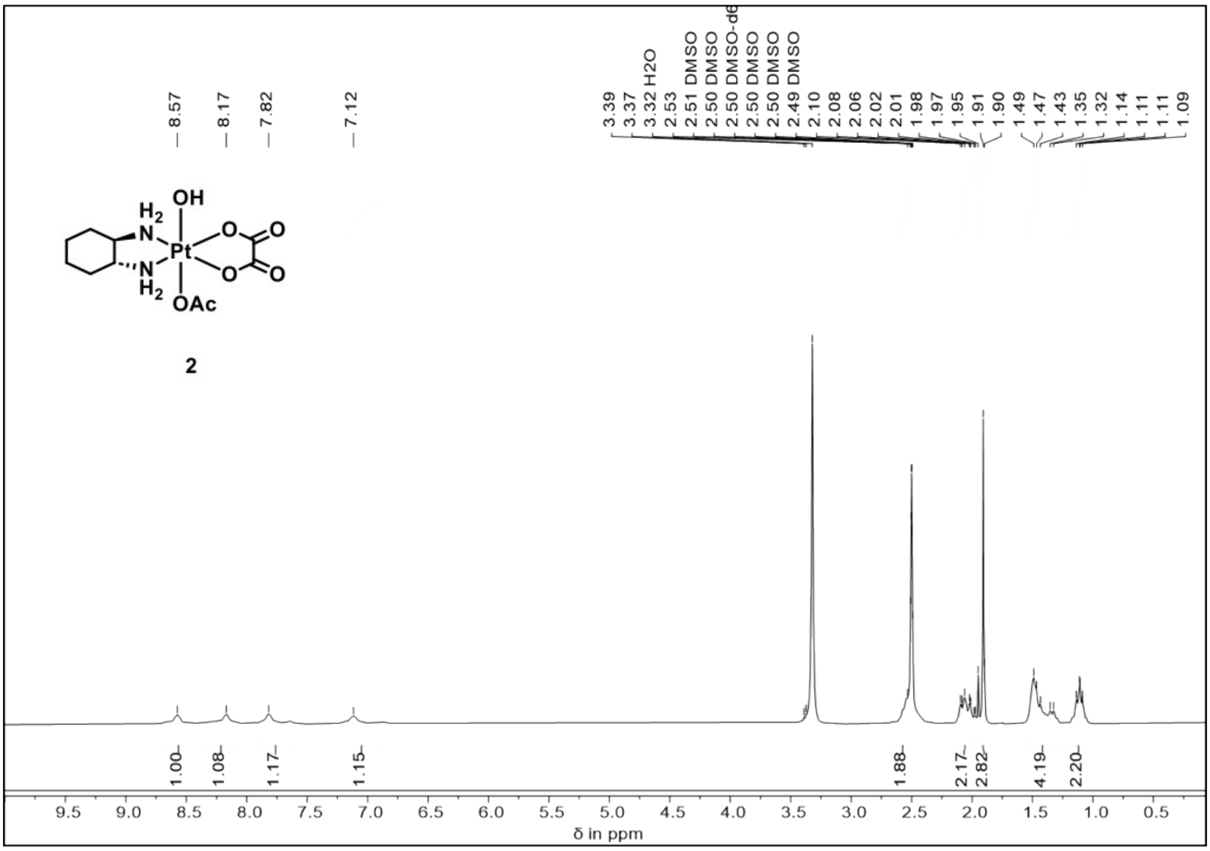

Figure S39. ^{1}H NMR (400 MHz, DMSO-d_6) of **Complex 2 [Pt(DACH)(ox)(OH)(OAc)].**

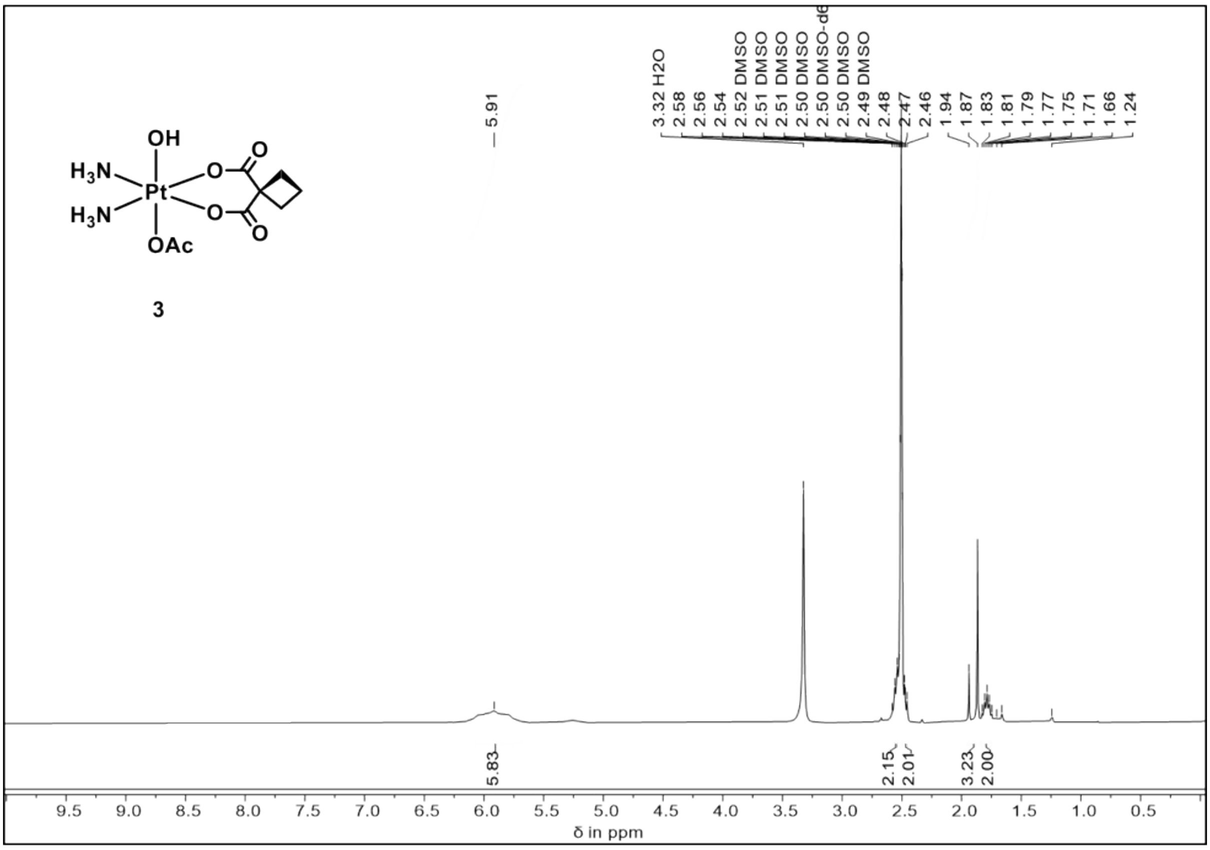

Figure S41. ^{1}H NMR (400 MHz, DMSO-d_6) of **Complex 3 [Pt(NH_3)$_2$(CBDCA)(OH)(OAc)].**

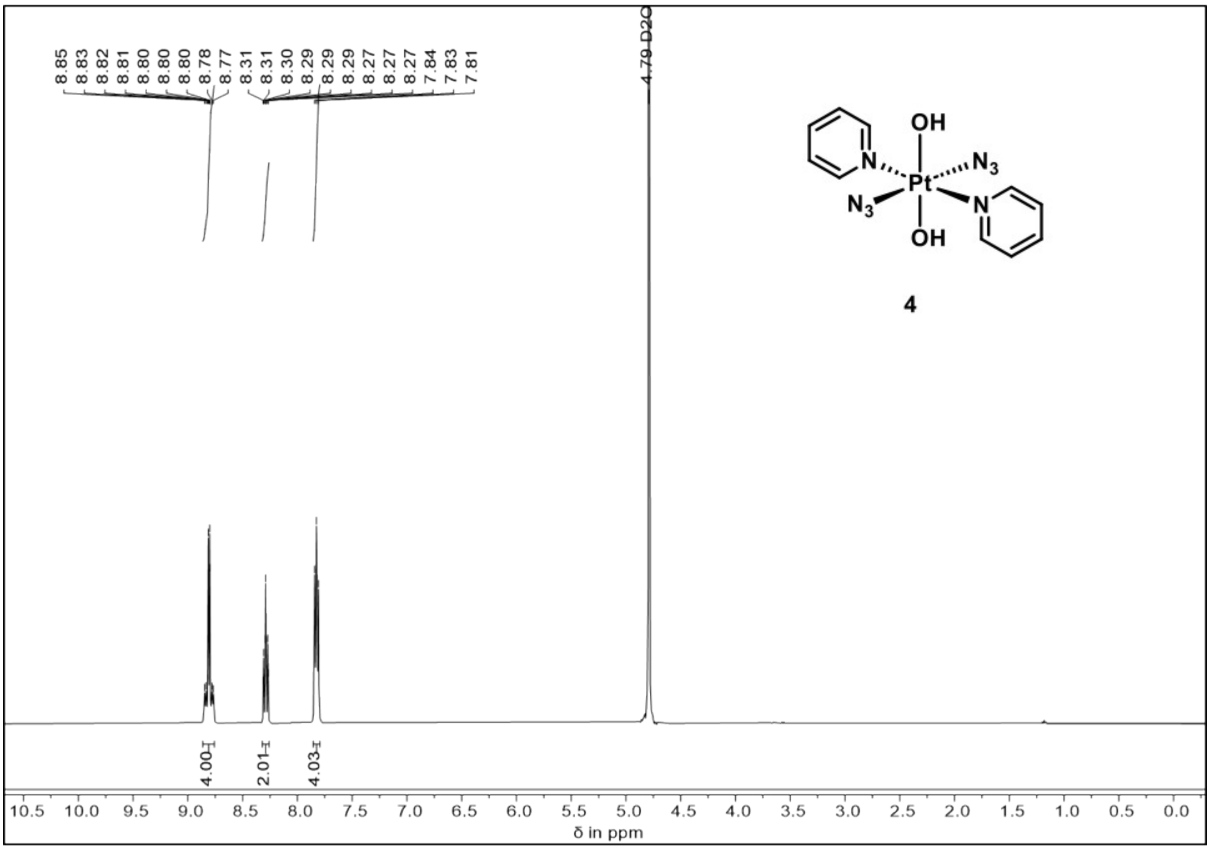

Figure S43. ^{1}H NMR (400 MHz, D_2O) of **Complex 4 *t,t,t*-[Pt(N_3)$_2$(py)$_2$(OH)$_2$].**

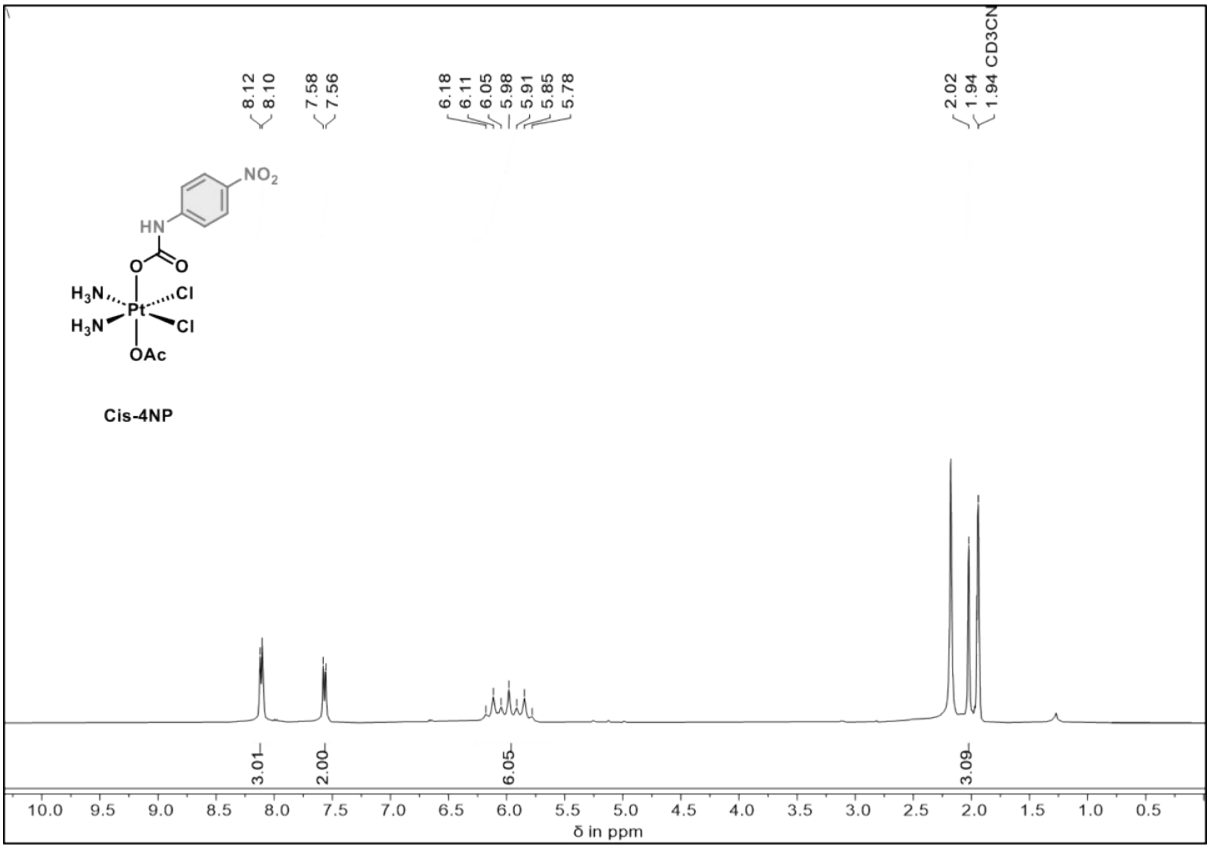

Figure S45. ^{1}H NMR (400 MHz, CD_3CN) of **Cis-4NP $[Pt(NH_3)_2Cl_2(OOCNHC_6H_4NO_2)(OAc)]$.**

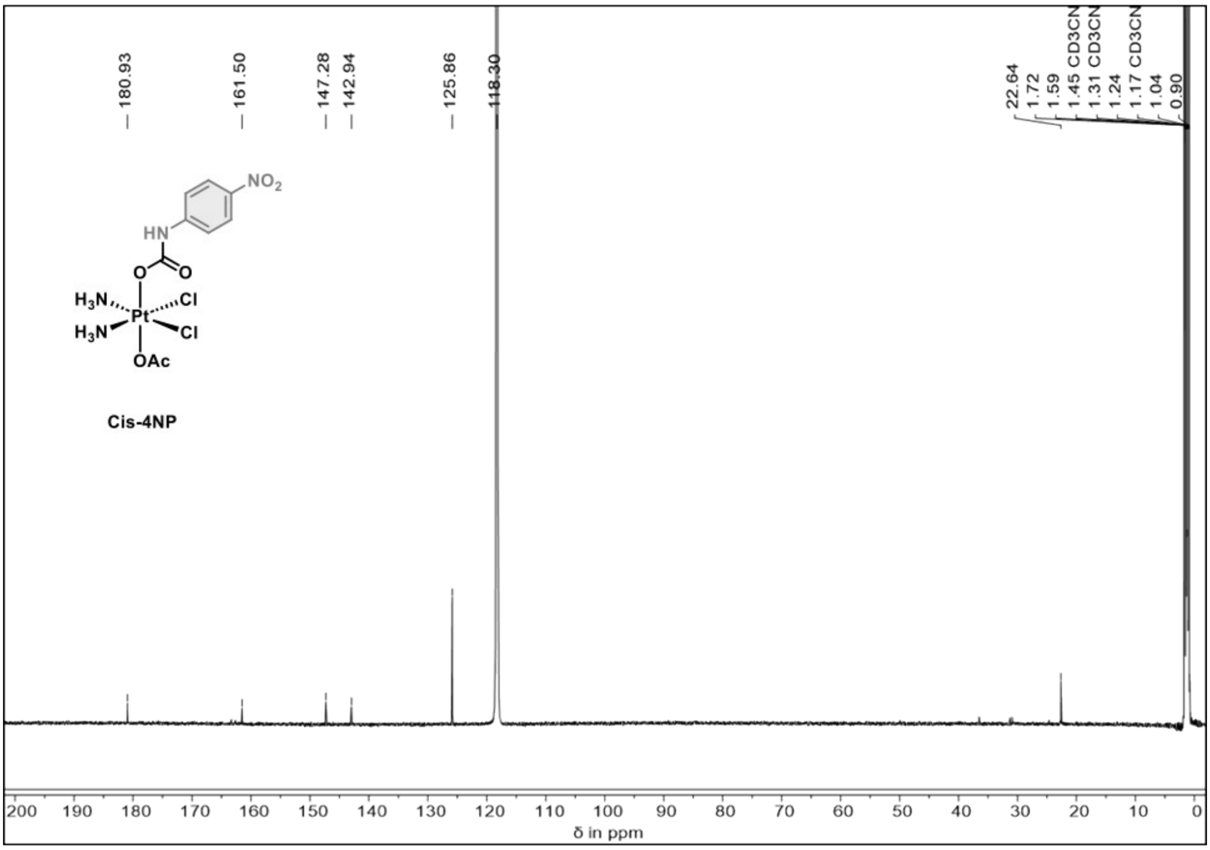

Figure S46. ^{13}C NMR (400 MHz, CD_3CN) of **Cis-4NP [$Pt(NH_3)_2Cl_2(OOCNHC_6H_4NO_2)(OAc)$].**

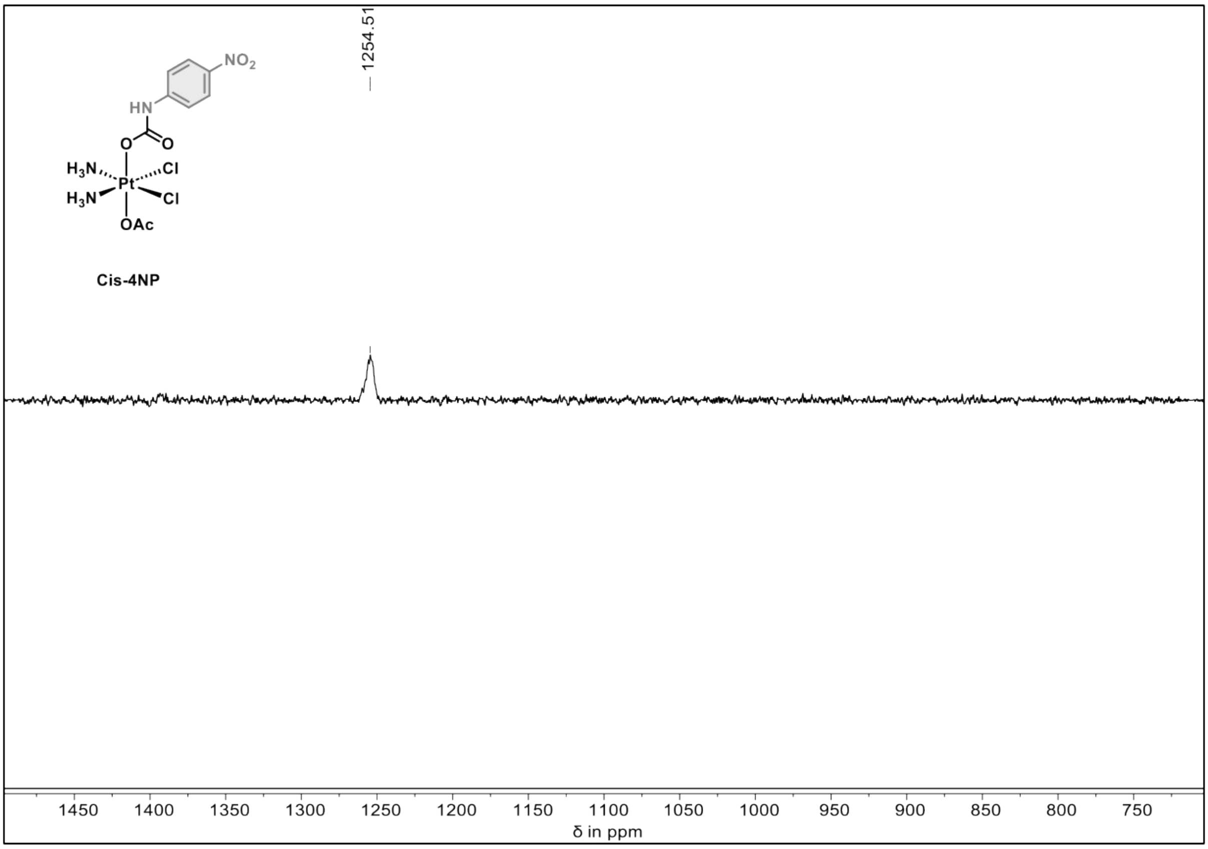

Figure S47. ^{195}Pt NMR (500 MHz, DMSO-d_6) of **Cis-4NP [Pt(NH$_3$)$_2$Cl$_2$(OOCNHC$_6$H$_4$NO$_2$)(OAc)].**

Expanded Spectrum RT 0.19, NL 2285682.25, Peak [1], Target Mass 540.0011

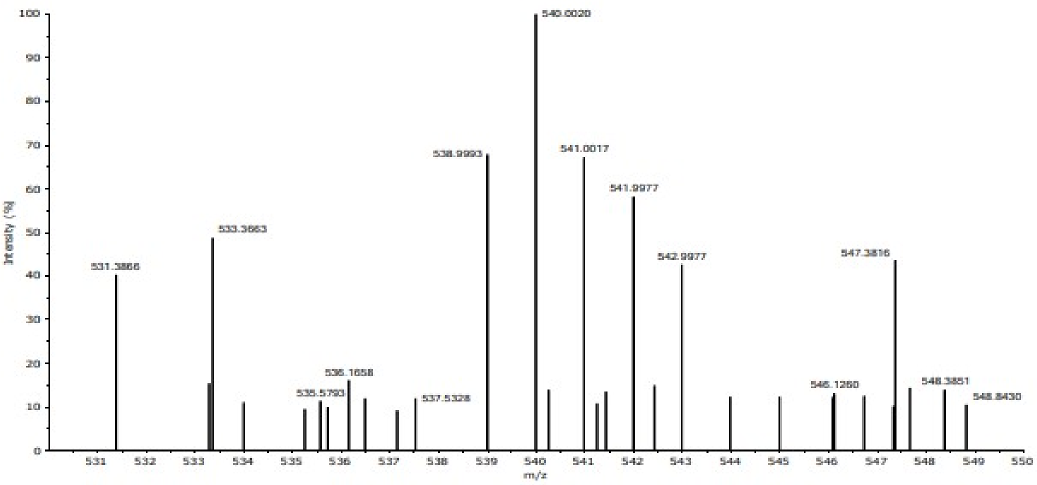

Theoretical Spectrum for C9H15Cl2N4O6Pt, Minimum Abundance 0.01%

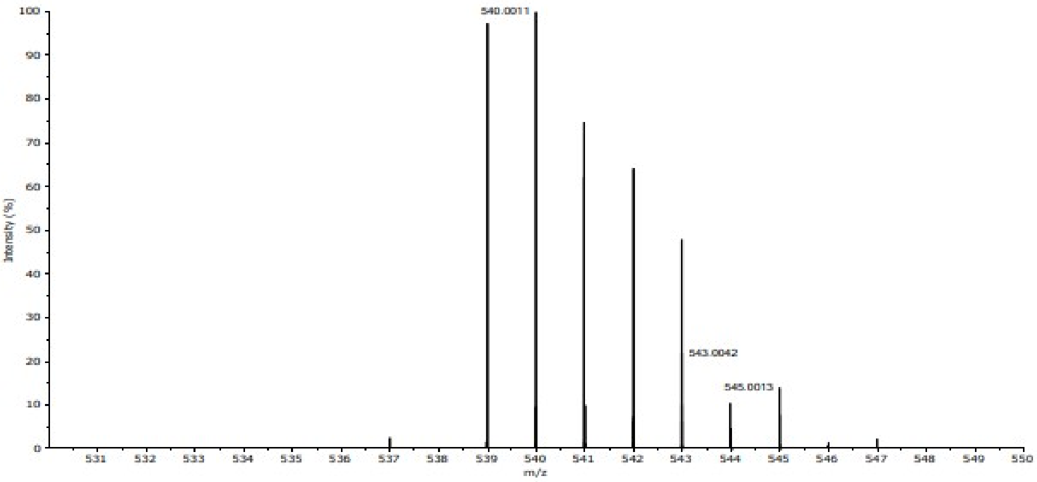

Measured Mass	Calculated Mass	Error (mDa)	Error (ppm)	Formula $[M+H]^+$	Response
540.0020	540.0011	0.91	1.68	$C_9H_{15}Cl_2N_4O_6Pt$	64943

The measured m/z value is consistent with your proposed formula for this sample.

Figure S48. HRMS of **Cis-4NP [Pt(NH$_3$)$_2$Cl$_2$(OOCNHC$_6$H$_4$NO$_2$)(OAc)].**

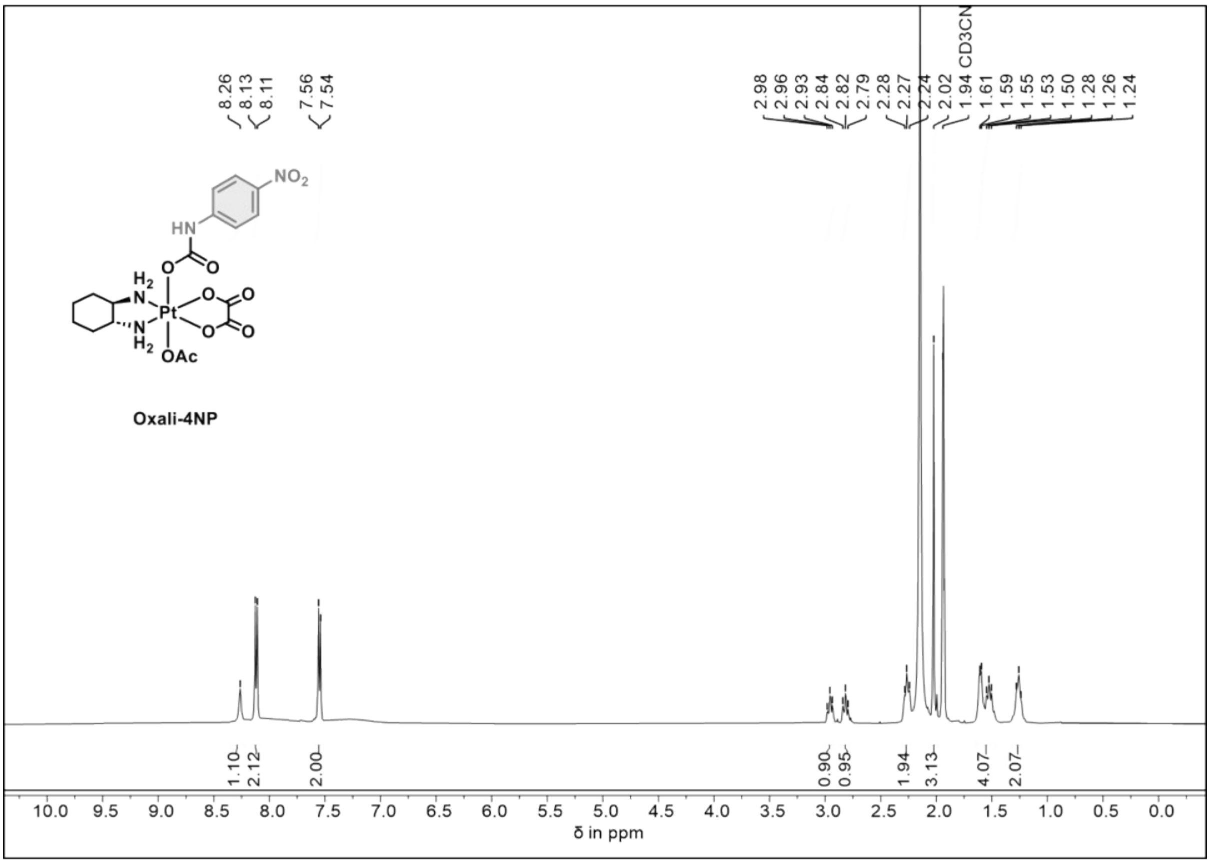

Figure.S49. ^{1}H NMR (400 MHz, CD_3CN) of **Oxali-4NP [Pt(DACH)(ox)($OOCNHC_6H_4NO_2$)(OAc)].**

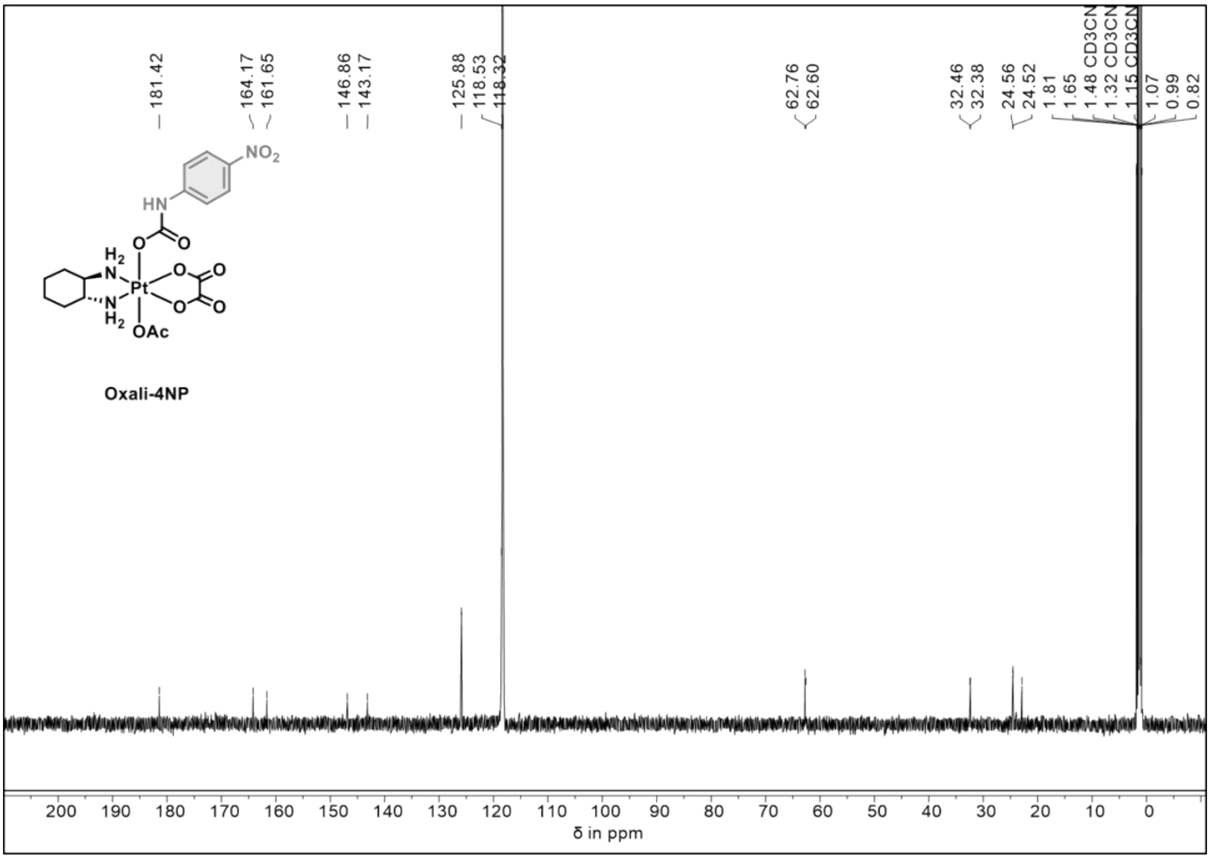

Figure S50. ^{13}C NMR (400 MHz, CD_3CN) of **Oxali-4NP [Pt(DACH)(ox)($OOCNHC_6H_4NO_2$)(OAc)].**

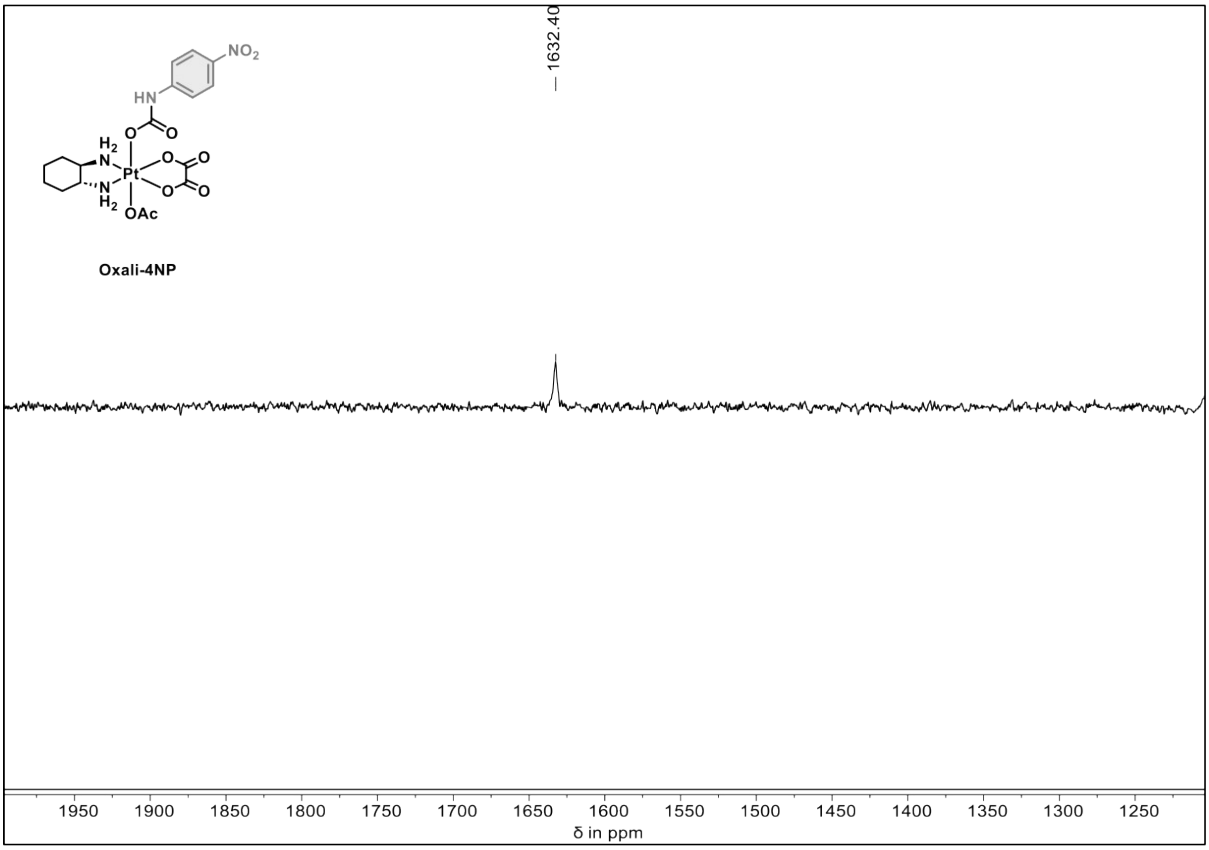

Figure S51. ^{195}Pt NMR (500 MHz, DMSO-d$_6$) of **Oxali-4Np [Pt(DACH)(ox)(OOCNHC$_6$H$_4$NO$_2$)(OAc)].**

Expanded Spectrum RT 0.20, NL 6179359, Peak [1], Target Mass 638.1057

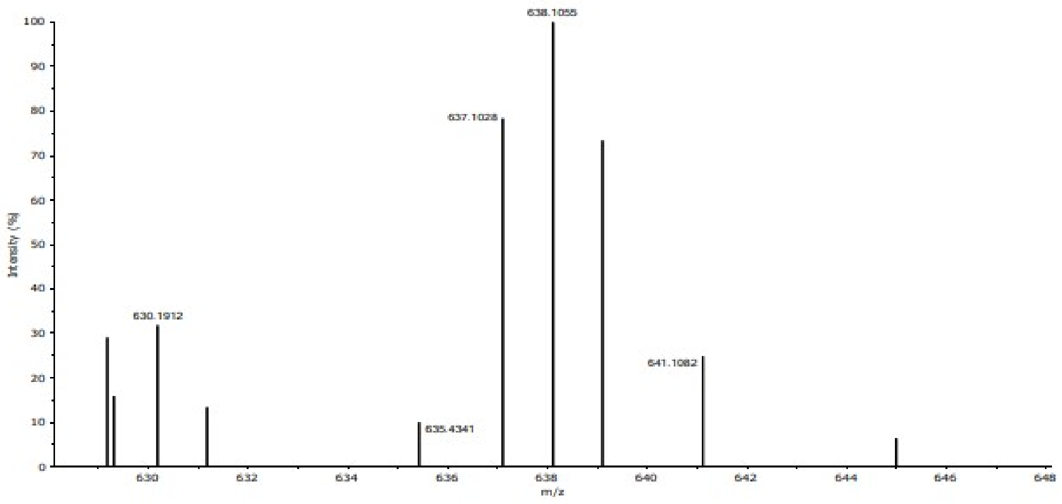

Theoretical Spectrum for C17H23N4O10Pt, Minimum Abundance 0.01%

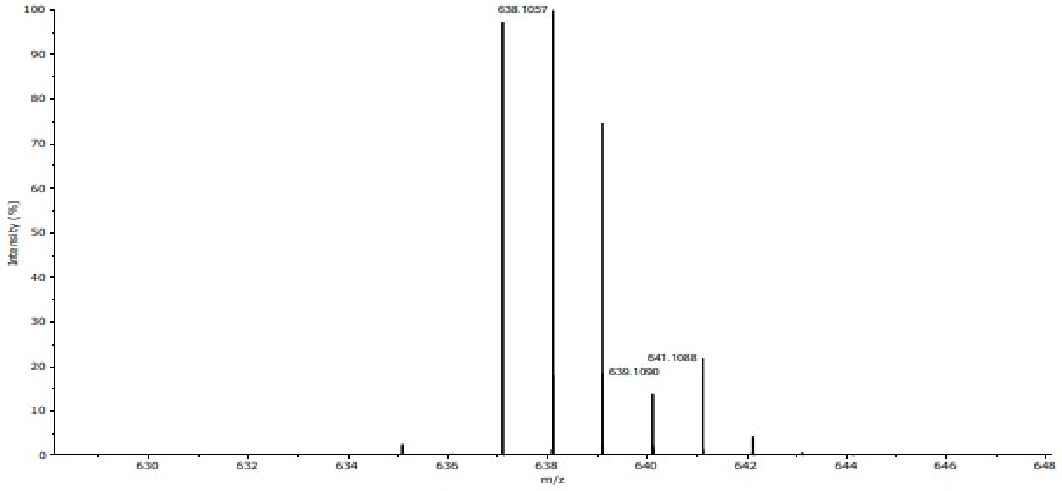

Measured Mass	Calculated Mass	Error (mDa)	Error (ppm)	Formula $[M+H]^+$	Response
638.1055	638.1057	-0.19	-0.30	$C_{17}H_{23}N_4O_{10}Pt$	314922

The measured m/z value is consistent with your proposed formula for this sample.

Figure S52. HRMS of **Oxali-4Np [Pt(DACH)(ox)(OOCNHC$_6$H$_4$NO$_2$)(OAc)].**

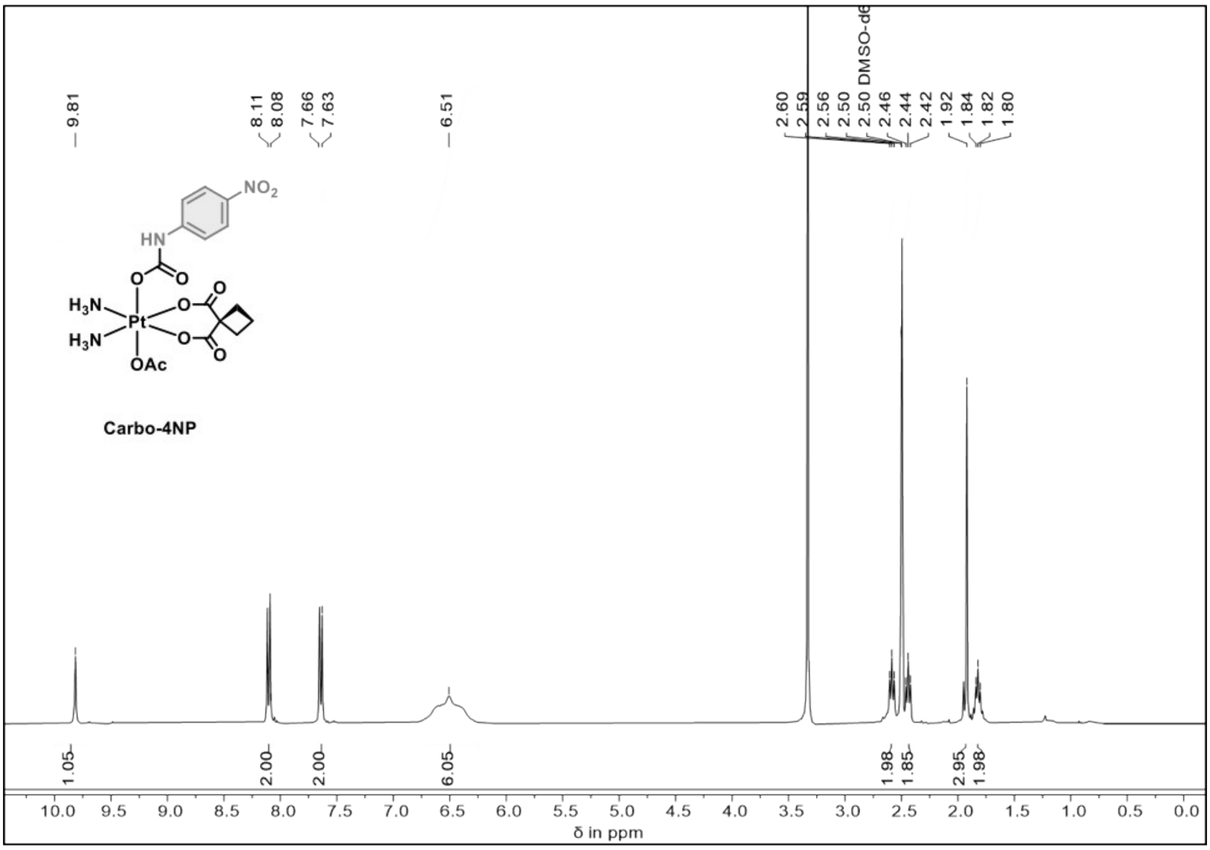

Figure S53. ^{1}H NMR (400 MHz, DMSO-d_6) of **Carbo-4NP [Pt(NH_3)$_2$(CBDCA)($OOCNHC_6H_4NO_2$)(OAc)].**

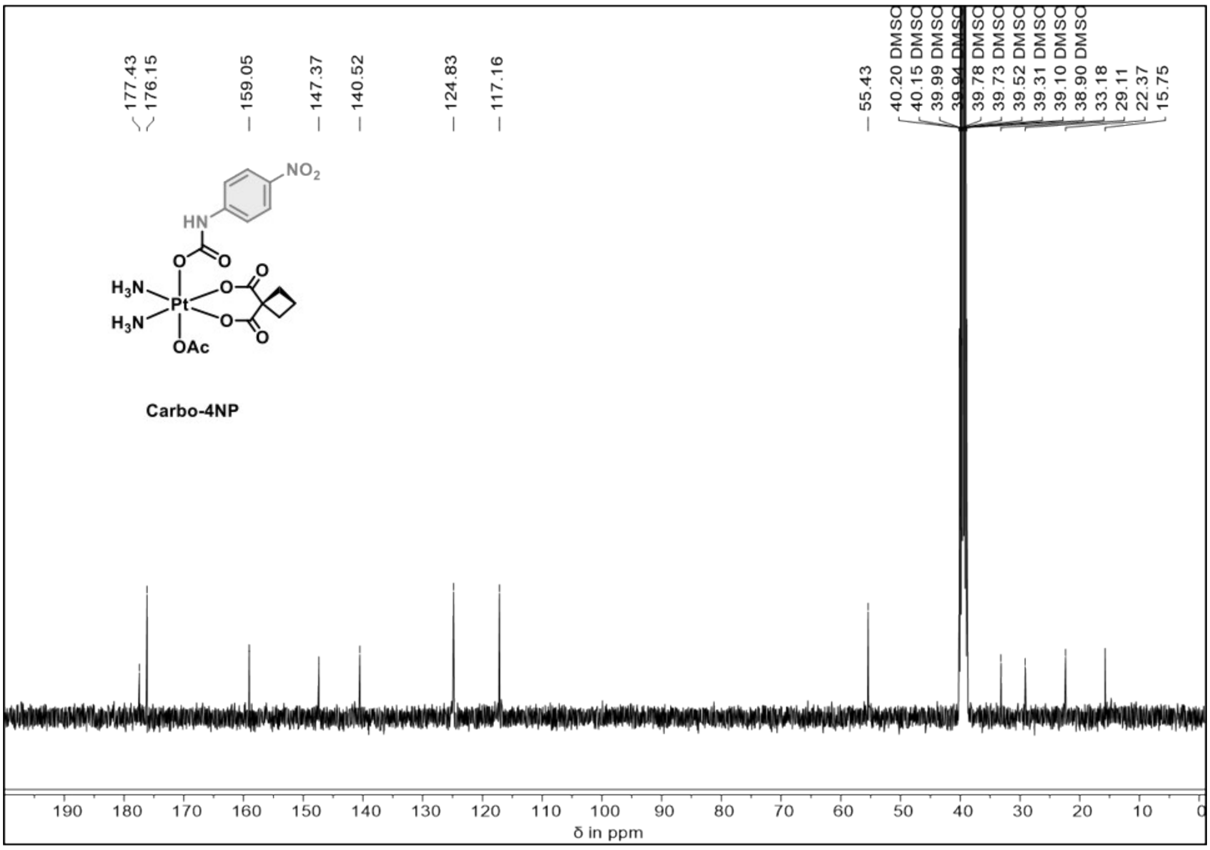

Figure S54. ^{13}C NMR (400 MHz, DMSO-d_6) of **Carbo-4NP [Pt(NH$_3$)$_2$(CBDCA)(OOCNHC$_6$H$_4$NO$_2$)(OAc)].**

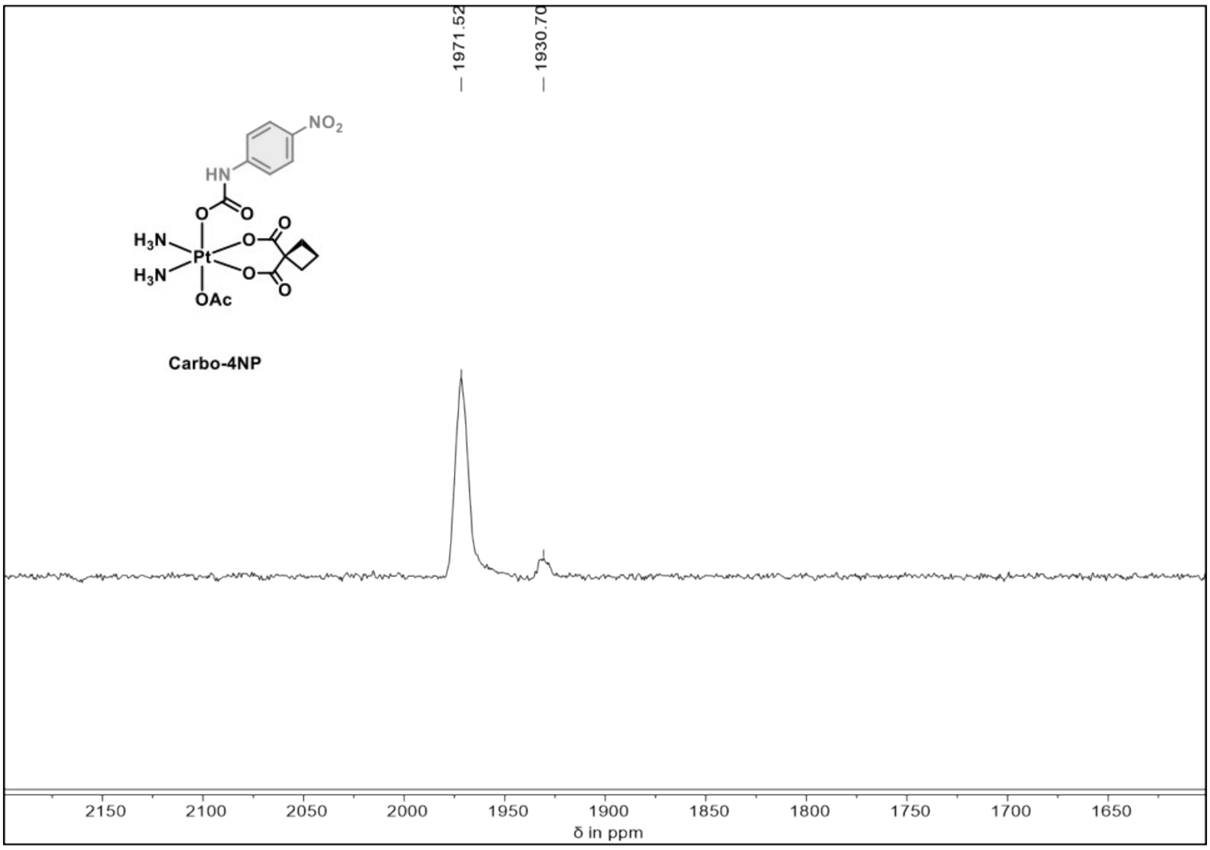

Figure S55. ^{195}Pt NMR (500 MHz, DMSO-d_6) of **Carbo-4NP [Pt(NH$_3$)$_2$ (CBDCA)(OOCNHC$_6$H$_4$NO$_2$) (OAc)].**

Expanded Spectrum RT 0.19, NL 1782867.5, Peak [1], Target Mass 612.0900

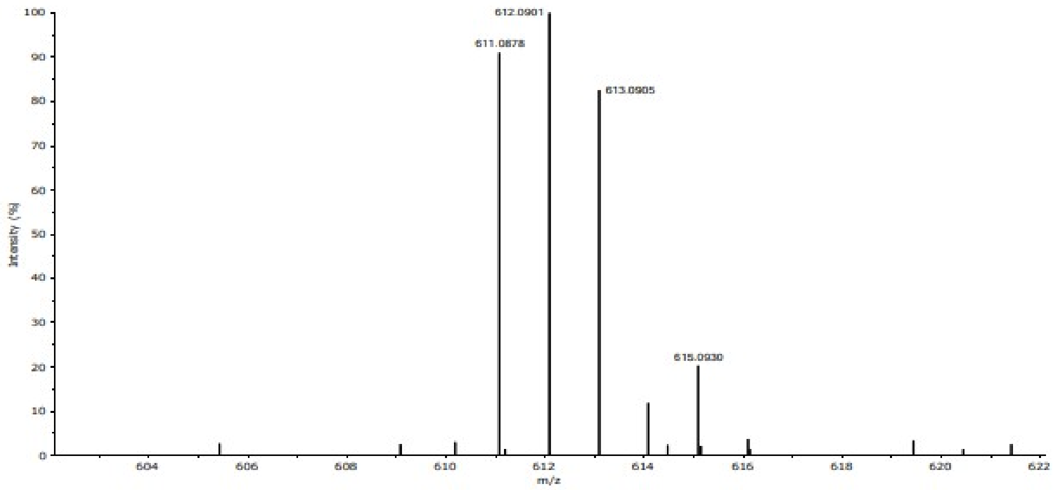

Theoretical Spectrum for C15H21N4O10Pt, Minimum Abundance 0.01%

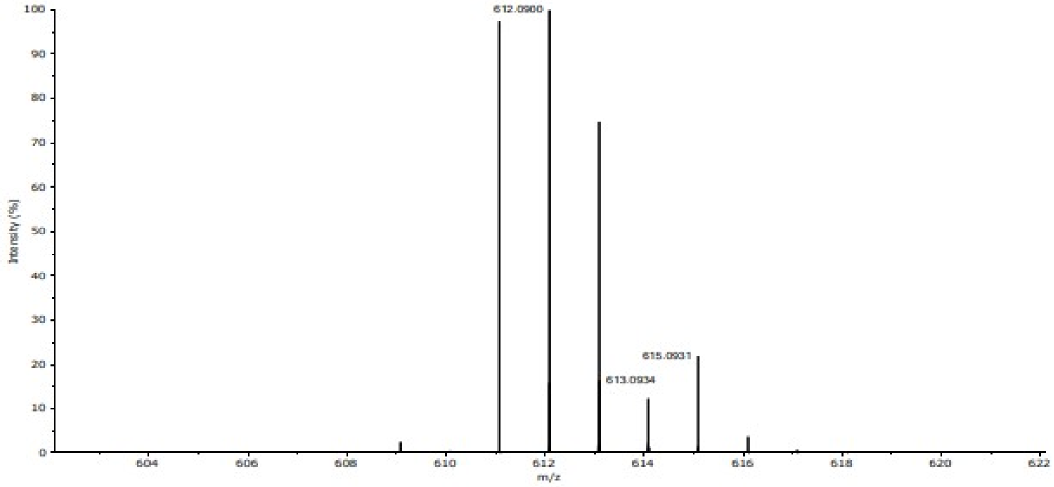

Measured Mass	Calculated Mass	Error (mDa)	Error (ppm)	Formula $[M+H]^+$	Response
612.0901	612.0900	0.08	0.12	$C_{15}H_{21}N_4O_{10}Pt$	1173482

The measured m/z value is consistent with your proposed formula for this sample.

Figure S56. HMRS of **Carbo-4NP $[Pt(NH_3)_2(CBDCA)(OOCNHC_6H_4NO_2)(OAc)]$.**

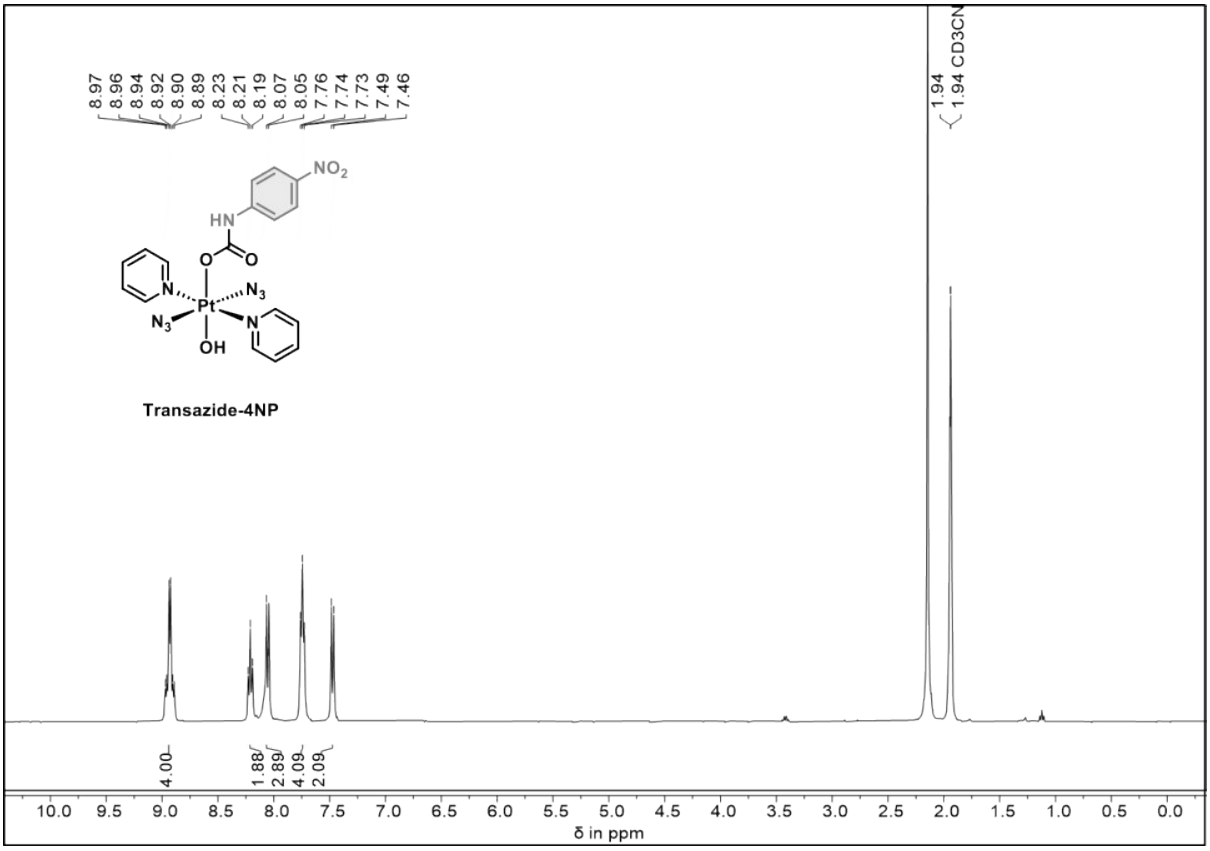

Figure S57. ^{1}H NMR (400 MHz, CD_3CN) of **Transazide-4NP t,t,t-[Pt(N_3)$_2$ (py)$_2$ ($OOCNHC_6H_4NO_2$)OH].**

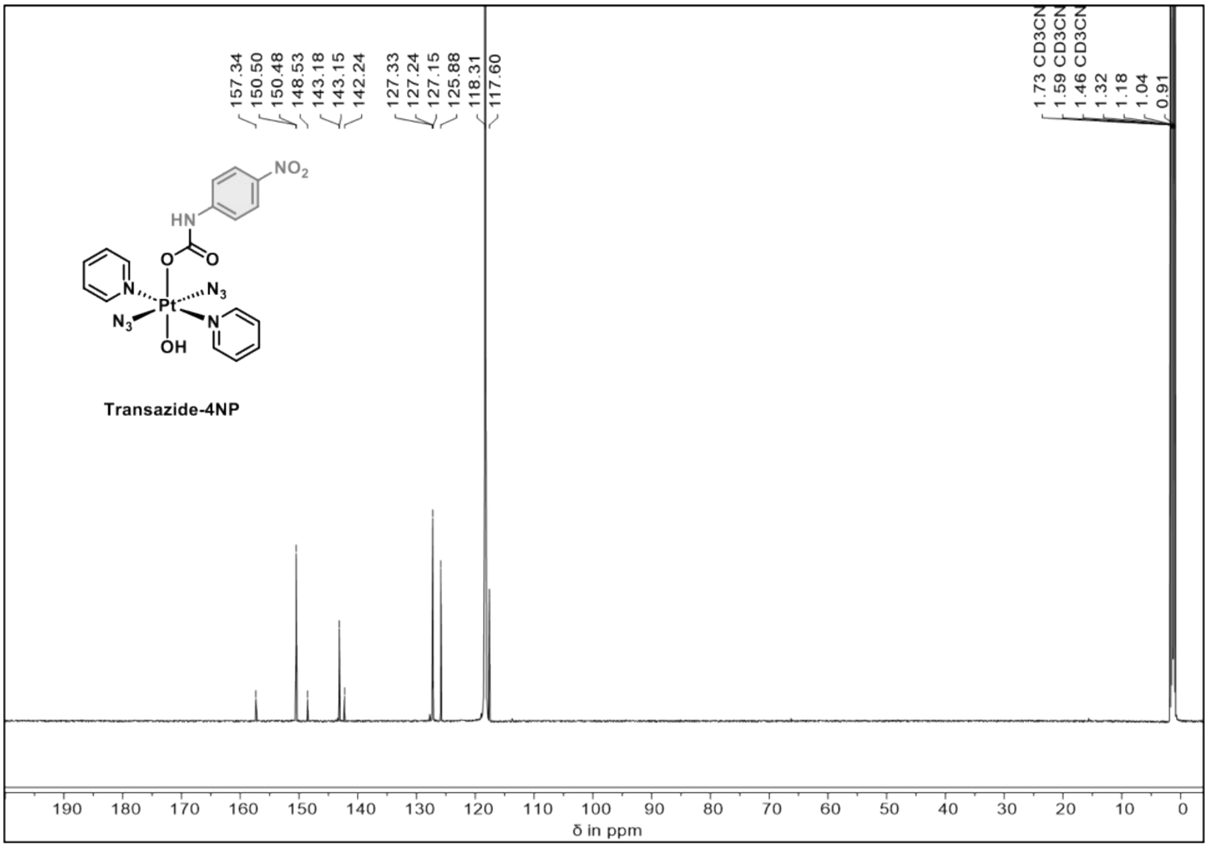

Figure S58. ^{13}C NMR (500 MHz, CD_3CN) of **Transazide-4NP t,t,t-[Pt(N_3)$_2$ (py)$_2$ ($OOCNHC_6H_4NO_2$)OH)].**

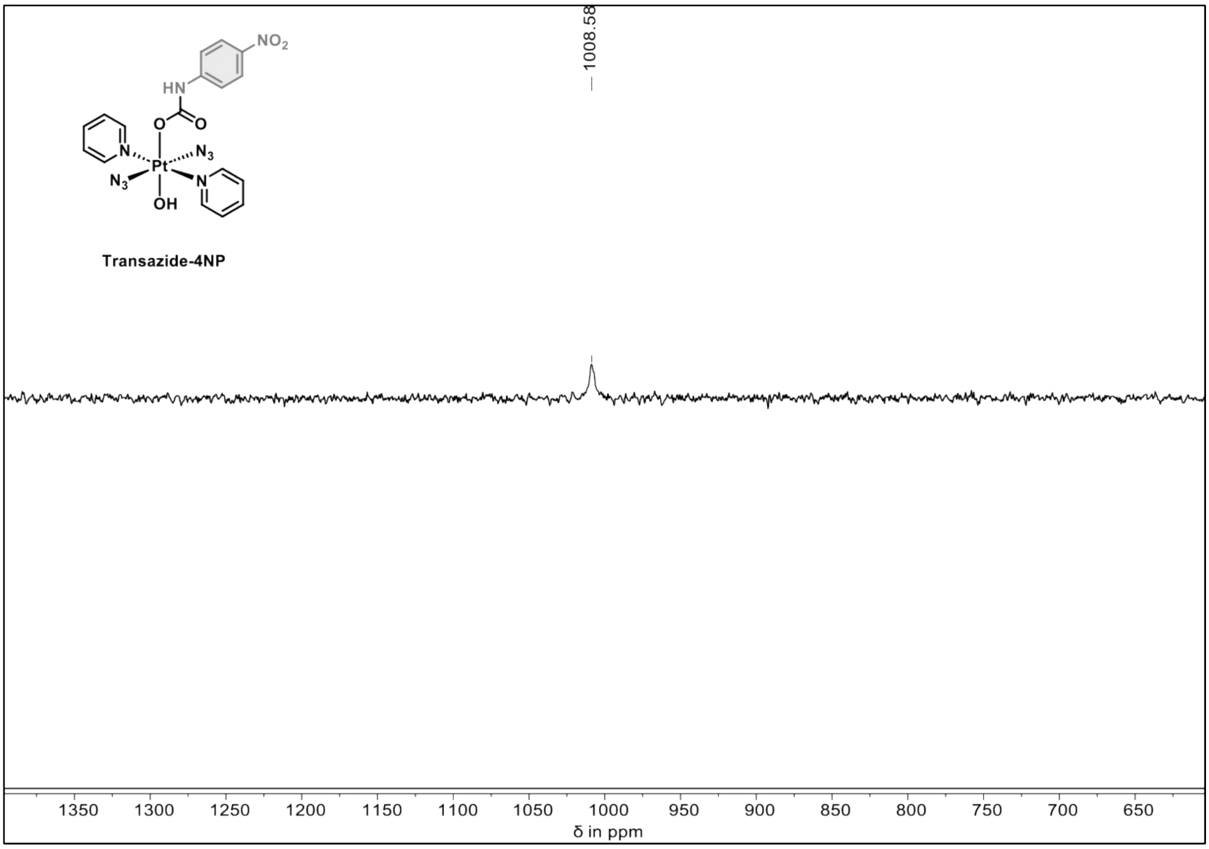

Figure S59. ^{195}Pt NMR (500 MHz, DMSO-d_6) of **Transazide-4NP t,t,t-[Pt(N_3)$_2$ (py)$_2$ (OOCNHC$_6$H$_4$NO$_2$)OH)].**

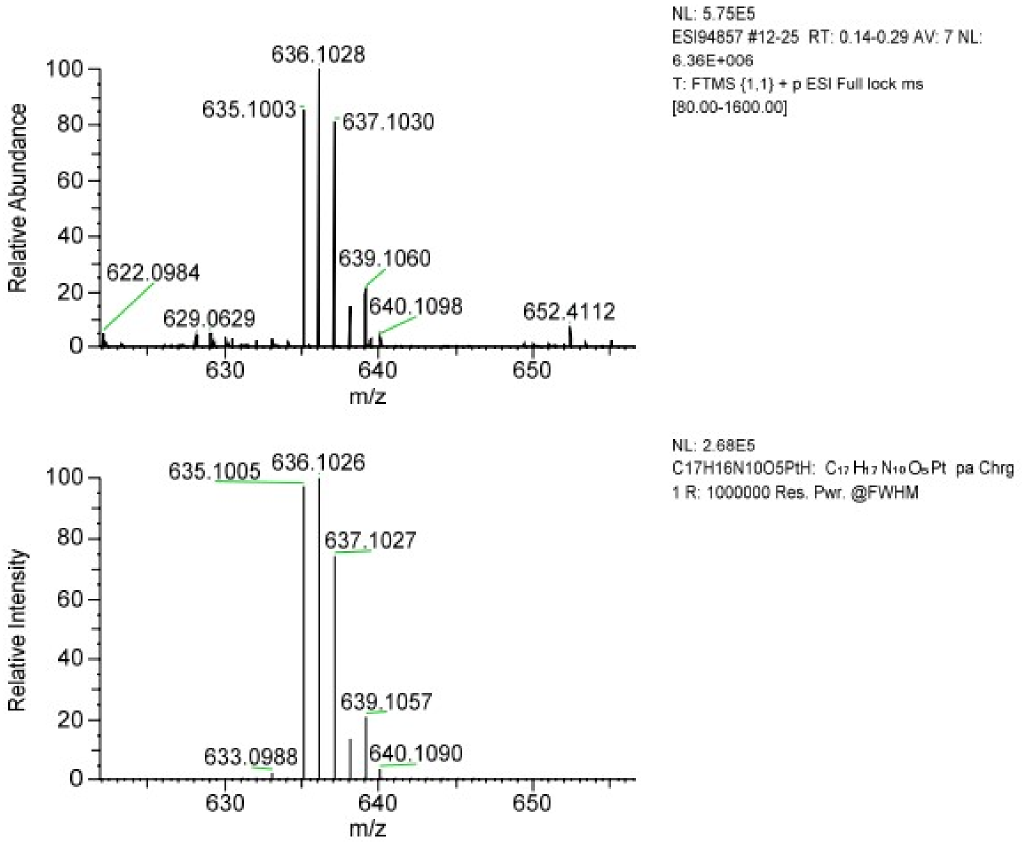

Mass accuracy 0.3 ppm.

Figure S60. HRMS of **Transazide-4NP t,t,t-[Pt(N_3)$_2$(py)$_2$ (OOCNHC$_6$H$_4$NO$_2$)OH]).**

6. References

1. H. Yao, Z. Xu, C. Li, M.-K. Tse, Z. Tong and G. Zhu, *Inorg. Chem.*, 2019, **58**, 11076–11084.

2. N. S. Sommerfeld, M. Hejl, M. H. Klose, E. Schreiber-Brynzak, A. Bileck, S. M. Meier, C. Gerner, M. A. Jakupec, M. S. Galanski and B. K. Keppler, *Eur. J. Inorg. Chem.*, 2016, **2017**, 1713–1720.

3. H. Yao, Y. F. Gunawan, G. Liu, M.-K. Tse and G. Zhu, *Dalton Trans.*, 2021, **50**, 13737–13747.

4. N. J. Farrer, J. A. Woods, L. Salassa, Y. Zhao, K. S. Robinson, G. Clarkson, F. S. Mackay and P. J. Sadler, *Angew. Chem., Int. Ed.*, 2010, **49**, 8905–8908.
